# *Myc* promotes polyploidy in murine trophoblast cells and suppresses senescence

**DOI:** 10.1242/dev.201581

**Published:** 2023-06-06

**Authors:** Vijay Pratap Singh, Huzaifa Hassan, Fengyan Deng, Dai Tsuchiya, Sean McKinney, Kevin Ferro, Jennifer L. Gerton

**Affiliations:** ^1^ Stowers Institute for Medical Research, Kansas City, MO 64110, USA; ^2^ Department of Biochemistry and Molecular Biology, University of Kansas Medical Center, Kansas City, KS 66160, USA

**Keywords:** Placenta, DNA damage, Polyploidy, Senescence, *Myc*, MTOR, RDNA, Nucleoli, Cytokine signaling, DNA replication

## Abstract

The placenta is essential for reproductive success. The murine placenta includes polyploid giant cells that are crucial for its function. Polyploidy occurs broadly in nature but its regulators and significance in the placenta are unknown. We have discovered that many murine placental cell types are polyploid and have identified factors that license polyploidy using single-cell RNA sequencing. *Myc* is a key regulator of polyploidy and placental development, and is required for multiple rounds of DNA replication, likely via endocycles, in trophoblast giant cells. Furthermore, MYC supports the expression of DNA replication and nucleotide biosynthesis genes along with ribosomal RNA. Increased DNA damage and senescence occur in trophoblast giant cells without *Myc*, accompanied by senescence in the neighboring maternal decidua. These data reveal *Myc* is essential for polyploidy to support normal placental development, thereby preventing premature senescence. Our study, combined with available literature, suggests that *Myc* is an evolutionarily conserved regulator of polyploidy.

## Teaser

**Highlighted Article:** Polyploidy is widespread in murine placental cell types. The transcription factor MYC supports polyploidy and prevents premature senescence in trophoblast giant cells.

## INTRODUCTION

The placenta is essential for the successful pregnancy of eutherian mammals (Burton and Fowden, 2015; Roberts et al., 2016). It performs several vital functions such as nutrient transport, hormone production and hematopoiesis, and protects the fetus from the immunological response of the mother (Carter, 2012; Haggarty et al., 2002; Rossant and Cross, 2001). Placental abnormalities caused by genetic and environmental insults can lead to fetal growth restriction, preterm birth and fetal death (Burton and Jauniaux, 2011; Burton et al., 2017; Hubel, 1999). Placental development starts from the trophectoderm of the blastocyst (Woods et al., 2018). The cells of the trophectoderm invade the endometrium of the uterus for the implantation of the blastocyst. The maternally derived decidua is in direct contact with the trophectoderm of the blastocyst (Ashary et al., 2018). Trophectoderm differentiates into several cell types, including primary and secondary trophoblast giant cells (parietal-TGCs), spongiotrophoblasts (SpTs) and glycogen cells (GlyTs), which together form the junctional zone of the placenta, which has an essential role in the production of several hormones (Georgiades et al., 2002; Simmons et al., 2007). The labyrinth zone, consisting of syncytiotrophoblasts, sinusoidal trophoblast giant cells (S-TGCs) and other cell types, is essential for nutrient and gas exchange. Parietal trophoblast giant cells (P-TGCs) and syncytiotrophoblasts are highly polyploid, meaning each cell contains multiple copies of the genome. The human placenta is morphologically distinct from the murine placenta, but also exhibits polyploidy (Simmons et al., 2007; Velicky et al., 2018).

Polyploidy is common throughout evolution from protozoans to mammals (Øvrebø and Edgar, 2018). The benefits of polyploidy have been proposed to be large cell size coupled with high metabolic activity and robustness to environmental stress (Schoenfelder and Fox, 2015). Cell fusion, endocycles and endomitosis are the three main mechanisms by which cells can increase ploidy (Fox and Duronio, 2013). Placental syncytiotrophoblasts exemplify cell fusion in which the cytoplasm of multiple cells fuses to form a multinucleated cell. Trophoblast giant cells likely form via endocycling, in which chromosomes undergo many rounds of DNA replication without cell division (Cross, 2005). In the case of endomitosis, cells go through early M phase but chromosome segregation does not occur, with megakaryocytes as a prime example (Ravid et al., 2002). Syncytiotrophoblasts and trophoblast giant cells have been identified as polyploid cells (Simmons et al., 2007). Although several placental cell types are recognized as oversized, their ploidy has not been carefully examined and polyploidy licensing factors are unknown.

In this study, we show that many murine placental cell types are polyploid, suggesting that the placenta is a highly polyploid organ. We demonstrate *Myc* (previously known as *c-Myc*) is a crucial regulator of polyploidy in placental development. *Myc* knockout mice illustrate *Myc* is essential to achieve polyploidy in trophoblast giant cells. MYC has known roles in regulating transcription by RNA polymerases I and II (Grandori et al., 2005; Rahl et al., 2010), consistent with our observations that MYC facilitates gene expression programs supporting DNA replication and rRNA production in trophoblast giant cells. Furthermore, without *Myc*, trophoblast giant cells accumulate DNA damage, activate cytokine signaling and senesce. Our results demonstrate the essential role of *Myc* in placental development and protection from premature senescence. Polyploidy depends on *Myc* in several *D. melanogaster* cell types*,* suggesting the role of *Myc* in promoting polyploidy has been conserved over ∼600 million years of evolution and preceded the development of the placental organ (Maines et al., 2004; Pierce et al., 2004; Qian et al., 2020).

## RESULTS

### Placental cell types maintain differing levels of polyploidy

**Fig. 1.**
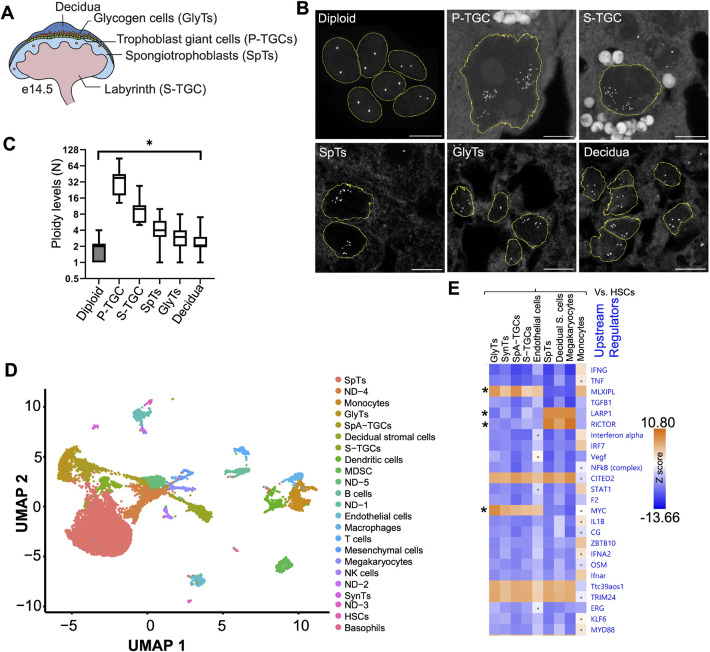
**Many placental cells are polyploid and show alterations in the *Myc* and inflammatory pathways.** (A) Schematic of the fully developed mouse placenta with different layers and cells. (B) DNA fluorescence *in situ* hybridization using 5S rDNA as a probe to show the number of copies of the genome in various placental cell types at 14.5 dpc using 10 µm paraffin wax-embedded sections. These are the maximum projection from *z* stacks. Trophoblast stem cells were used as diploid controls. *n*=3 biological replicates. Scale bars: 10 µm. (C) Quantification of the number of 5S rDNA foci from different cell types. Diploid, *n*=17; P-TGCs, *n*=11; S-TGCs, *n*=8; SpTs, *n*=168; GlyTs, *n*=156; decidua, *n*=144). The box plot shows the ploidy of various cell types. In box plots, boxes represent interquartile range and whiskers represent minimum and maximum values. *P<0.05. (D) UMAP (uniform manifold approximation and projection) of 14.5 dpc placenta using scRNA-seq analysis. Each dot represents a single cell. Two biological replicates were performed. (E) Differential gene expression was performed between diploid (hematopoietic stem cells) and other polyploid cells. Ingenuity pathway analysis (IPA) using differentially expressed genes shows changes in upstream regulators in polyploid cells. Asterisks highlight the pathways differentially used by several placental cell types when compared with non-placental cells.

Fully developed murine placenta consists of several cell types (Simmons et al., 2007). The polyploidy of P-TGCs and syncytiotrophoblasts has been established, whereas the ploidy of other placental cell types has not been scrutinized (Dupressoir et al., 2009, 2011; Varmuza et al., 1988) (Fig. 1A). We employed DNA fluorescence *in situ* hybridization (DNA-FISH) using a 5S ribosomal RNA gene probe to determine the ploidy of placental cell types at 14.5 dpc in paraffin wax-embedded sections. To validate the method, we examined diploid trophoblast stem cells (Singh and Gerton, 2021) and we observed two distinct 5S signals per nucleus from the two homologs (Fig. 1B). Next, we performed DNA-FISH on placental sections and identified different cell types based on their morphology and spatial organization. As reported previously, P-TGCs are highly polyploid: >64C, as evident from many foci of 5S (Fig. 1B,C). Surprisingly, the placental cell types we analyzed, including S-TGCs, SpTs and GlyTs, had ploidy >8C based on multiple 5S foci (Fig. 1B,C). Interestingly decidual cells, which originate from the mother's endometrium, are also polyploid (Fig. 1B,C). Additional cell types could not be identified and evaluated without markers. Overall, the placental organ appears to be composed of many polyploid cell types.

5S foci are grouped into two loose clusters in P-TGCs, consistent with previous reports that sister chromatids remain associated (Varmuza et al., 1988). Interestingly, other polyploid cells show similar clustering, indicative of a common endocycle program. We also observed cells with one cluster, potentially due to the second cluster being in a different plane, a caveat of analyzing large nuclei in a single tissue slice. To further validate the clustering phenotype in P-TGCs, we used a DNA-FISH probe for the *Prl8a8* gene and observed a similar pattern of two loose clusters (Fig. S1A), extending the observation to a second genomic locus on a different chromosome. Unfortunately, the *Prl8a8* probe did not provide sufficient signal to quantify ploidy of other cell types (Fig. S1A). We tried probing another multicopy gene, 45S, but the gene arrays exist on five different chromosomes (Potapova et al., 2019) and we were not able to detect distinct clusters (Fig. S1B). This loose association and heterogeneity is distinct from *Drosophila* polytene chromosomes in the salivary gland, which form more homogeneous tight associations (Stormo and Fox, 2017) but show some similarities to *Drosophila* polyploid nurse cells and rectal papillar cells, where chromatids are loosely associated at different stages of their development (Fig. 1B and Fig. S1A) (Dej and Spradling, 1999; Stormo and Fox, 2016). Altogether, these data are the first to demonstrate widespread polyploidy in the murine placenta, and further suggest that this genomic status may confer important function(s).

### Polyploid cells show suppression of inflammatory pathways

To discover the molecular regulators of polyploidy in placental cells, we performed single-cell RNAseq (scRNAseq) using 10X Genomics technology from 14.5 dpc placenta. We generated a single cell suspension and captured a total of 39,399 cells from two placenta. UMAP analysis was performed to find the distance between the cells based on gene expression analysis. We were able to identify 34 clusters (Fig. S2A and Table S1). When we examined markers, we observed several clusters with hemoglobin expression, consistent with the presence of red blood cells (RBCs). Although we treated the cell suspension with RBC removal buffer, the placenta is a rich source of maternal and fetal blood cells, so RBCs are unavoidable. For further analysis, we removed cells with hemoglobin a and b expression, and clustered the remaining 16,856 cells. Twenty-eight clusters emerged (Fig. S2B and Table S1). Based on published markers, we were able to identify 23 cell types (Fig. 1D). Out of 23 cell types, seven were placental [SynTs (Marsh and Blelloch, 2020), GlyTs (Coan et al., 2006), SpA-TGCs (Nelson et al., 2016), S-TGCs (Simmons et al., 2008a), SpTs (Simmons et al., 2008b), HSCs (Okada et al., 1992) and endothelial cells (Nelson et al., 2016)], one was decidual [decidual stromal cells (Simmons et al., 2008b)], ten were blood lineage derived [T cells (Alarcon et al., 1988), B cells (Mason et al., 1995), NK cells (Fehniger et al., 2007), megakaryocytes (Bianchi et al., 2016), macrophages (Zhang et al., 2008), dendritic cells (Moore and Anderson, 2013), basophils (Torrero et al., 2009), monocytes (Sunderkötter et al., 2004), myeloid-derived suppressor cells (MDSC) (Zhao et al., 2012) and mesenchymal cells (Marsh and Blelloch, 2020)] and five (ND1- to ND-5) had undetermined identities (Fig. S2C). In each cluster, we were able to detect over 10,000 genes (Fig. S2D). We identified the majority of known placental cell types, except P-TGCs, because their large size causes their exclusion from the cell-sorting apparatus.

We compared the gene expression of diploid hematopoietic stem cells with other diploid and polyploid cells from hematopoietic and trophoblastic origin to discover polyploid-specific pathways. For diploid cells, we used hematopoietic stem cells and monocytes. For polyploid cells, we used GlyTs, SynTs, SpA-TGCs, S-TGCs, SpTs, decidual stromal cells and megakaryocytes (Hancock et al., 1993; Simmons et al., 2007). We identified the top 25 upstream regulators specific to polyploid cell types using ingenuity pathway analysis (Qiagen) (Fig. 1E). We found programs regulated by *Cited2*, *Trim24* and *Ttc39aos1* are activated in all polyploid cell types. *Cited2* encodes a transcriptional co-regulator (CREBBP/EP300) and has been shown to regulate placental development. In the absence of this gene, placentas are smaller due to a reduction in cell number (Imakawa et al., 2016; Withington et al., 2006). *Trim24* (tripartite motif containing 24) encodes a co-regulator of many nuclear receptors, is misregulated in many cancers (Herquel et al., 2011) and suppresses interferon γ (IFNG) Tisserand et al., 2011). *Ttc39aos1* encodes a long noncoding RNA (lincRNA–EPS) that binds PKR and antagonizes viral RNA-PKR interaction (Zhu et al., 2022). Interestingly the most repressed pathways in polyploid placental cells are related to inflammation, such as IFNG, TNF, interferon α, IRF7, NFκB, STAT1, IL1B, choriogonadotropin (CG or hCG), ZBTB10, IFNA2, OSM, Ifnar, KLF6 and MYD88 (Bayer and Alcaide, 2021; Furcron et al., 2016; Richards, 2013; Smita et al., 2021; Wan et al., 2007). These pathway signatures suggest that polyploid cell types share transcriptomic programs characterized by the suppression of immune response pathways compared with diploid cells. We speculate that the suppression of inflammatory pathways in polyploid placental cells may help the placenta and fetus co-exist with the mother's immune system.

### A group of polyploid placental cell types activates the *Myc* pathway

We searched for differential expression of pathways that could drive polyploidy in placental cells. Three upstream regulators, encoded by *Rictor*, *Larp1* and *Myc*, were differentially identified between distinct polyploid cell types (Fig. 1E). In mammals, the mTOR complex exists in two versions, mTOR1 (*Raptor*) and mTOR2 (*Rictor*) (Guertin et al., 2006). mTOR2 (*Rictor*) is active in megakaryocytes, decidual stromal cells and SpTs, whereas other polyploid cell types show repression of this pathway. Similarly, *Larp1*, which encodes an inhibitor of ribosomal protein mRNA transcription, appears to be active in megakaryocytes, decidual stromal cells and SpTs. mTOR1 activates ribosomal protein transcription by removing LARP1 from ribosomal protein mRNA. The pattern of upstream regulators suggests the mTOR1 (*Raptor*) pathway is repressed in megakaryocytes, decidual stromal cells and SpT polyploid cells (Fig. 1E) (Lahr et al., 2017), in contrast to its activation in a second group of polyploid cells that includes TGCs. *Myc* also showed a unique pattern of low expression in megakaryocytes, decidual stromal cells and SpTs but high in the same polyploid placental cell types that have active mTOR1. *Myc* has been suggested to play a significant role in placental development from previous studies and supports polyploidy in *D. melanogaster* (Dubois et al., 2008; Pierce et al., 2004; Shcherbata et al., 2004). We decided to characterize the role of *Myc* in ploidy regulation in the placenta.

### *Myc* regulates placental ploidy

**Fig. 2.**
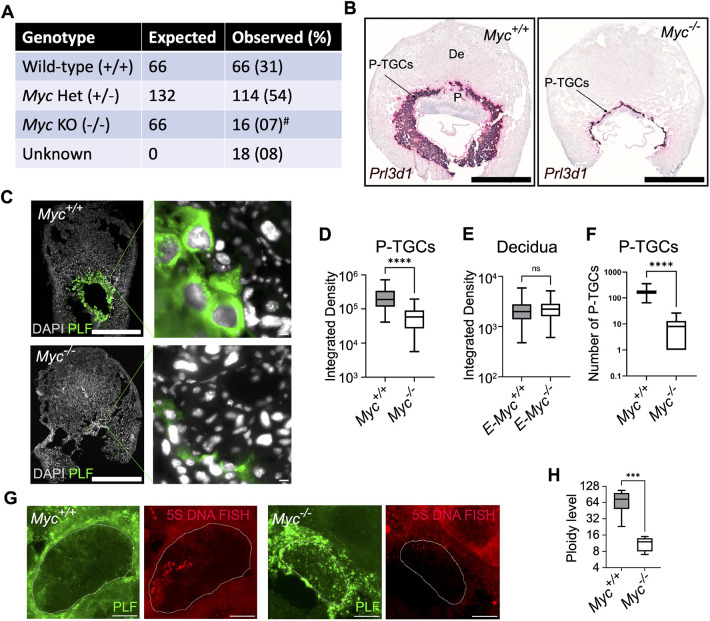
***Myc* is required for polyploidy in P-TGCs.** (A) The number of embryos collected from the cross of *Myc*^+/−^ male and *Myc* ^fl/+^:*Zp3*-Cre or *Myc*^+/−^ females at 9.5 dpc. *Myc*^−/−^ mice were born at a sub-mendelian ratio. ^#^*P*<0.05 (χ^2^ square test). (B) RNA FISH on paraffin wax-embedded sections using *Prl3d1*, a marker of P-TGCs showed reduced TGC layer and placenta size in the *Myc*^−/−^ genotype at 9.5 dpc. Nuclei were counterstained with Hematoxylin. *n*=3 or 4 biological replicates. Scale bars: 1 mm. (C) Placental cryosections from wild type and *Myc*^−/−^ at 9.5 dpc were stained using an anti-proliferin (PLF) antibody to mark P-TGCs. Nuclei were counterstained with DAPI. *n*=3 biological replicates. Scale bars: 1 mm; 10 µm (enlargements). (D) Box plot showing quantification of integrated DAPI intensity from P-TGCs of wild-type and *Myc*^−/−^ placenta at 9.5 dpc*. n*=66-84 nuclei. ****P<0.0001. (E) Box plot showing quantification of integrated DAPI intensity from decidual cells attached to wild-type and *Myc*^−/−^ placenta at 9.5 dpc*. n*=180-183 nuclei. (F) Box plot showing the numbers of P-TGCs, using *Prl3d1* as marker, in wild-type and *Myc*^−/−^ placenta at 9.5 dpc*. n*=6-8 biological replicates. ****P<0.0001 (unpaired Student's *t*-test). (G) DNA -FISH using 5S rDNA as a probe was used to analyze the ploidy of wild-type and *Myc*^−/−^ P-TGCs. These are the maximum projection from *z*-stacks. Scale bars: 10 µm. (H) Box plot showing quantification of 5S rDNA signal from wild-type and *Myc*^−/−^ P-TGCs. *n*=6 or 7 biological replicates. ***P<0.001 (unpaired Student's *t*-test). In box plots, boxes represent interquartile range and whiskers represent minimum and maximum values.

*Myc* is highly expressed in many placental cell types (Dubois et al., 2008). Placental deficiency accounts for the majority of developmental abnormalities and lethality observed in *Myc* knockout mice based on an embryo specific knockout (Dubois et al., 2008). Lack of placental development, including small size has been documented with deletion of *Myc* in the epiblast, with no significant difference in embryo size up to E10.5 (Dubois et al., 2008). We focused on the role of *Myc* in ploidy regulation in placental cells. We crossed *Myc* floxed mice with *Zp3*-Cre mice, to knockout the *Myc* gene in oocytes. We collected several litters at 9.5 dpc*.* Consistent with the literature, *Myc* null embryos were born at sub-mendelian ratios (Fig. 2A) (Davis et al., 1993). *Myc* null embryos were observed at 7%, compared with the expected frequency of 25% (*P*<0.05). 8% of the embryos were too small for genotyping, consistent with previous reports that loss of *Myc* reduces embryo size via placental defects (Dubois et al., 2008). If we assume these are null and combine them with the genotyped null embryos, this number is still well below the expected frequency (*P*<0.05). To determine the efficiency of Cre mediated *Myc* deletion, we analyzed MYC protein expression in P-TGCs, marked with proliferin, at 9.5 dpc. MYC expression was not detected in *Myc* null P-TGCs (Fig. S3). Taken together, these data demonstrate that *Myc* is required for embryonic viability.

Next, we characterized placental development in histological sections. We performed RNA *in situ* hybridization for *Prl3d1* to mark P-TGCs (Simmons et al., 2008b). We observed the formation of chorioallantoic placenta with a hypoplastic labyrinth layer and reduction in size associated with *Myc*-null embryos, as expected (Dubois et al., 2008) (Fig. 2B). P-TGCs formed but the number and size were drastically reduced (Fig. 2C,D,F). To further understand the mechanism of size reduction, we analyzed the ploidy of P-TGCs (Fig. 2C). DAPI integrated density was measured for P-TGCs identified by proliferin expression. Nuclear size was almost fourfold smaller in *Myc*-null P-TGCs when compared with wild type (Fig. 2D). As a control, we compared nuclear size for decidual cells, which are derived from the endometrium of the mother and therefore contain at least one copy of *Myc*. The nuclear size was unchanged (Fig. 2E). We further analyzed ploidy in P-TGCs by DNA-FISH using a 5S ribosomal DNA probe. We observed a sixfold reduction in ploidy in *Myc*-null P-TGCs when compared with wild type (Fig. 2G,H). These results suggest that, in the absence of *Myc*, the ploidy of P-TGCs is severely reduced.

### *Myc* regulates replication and inflammation genes

**Fig. 3.**
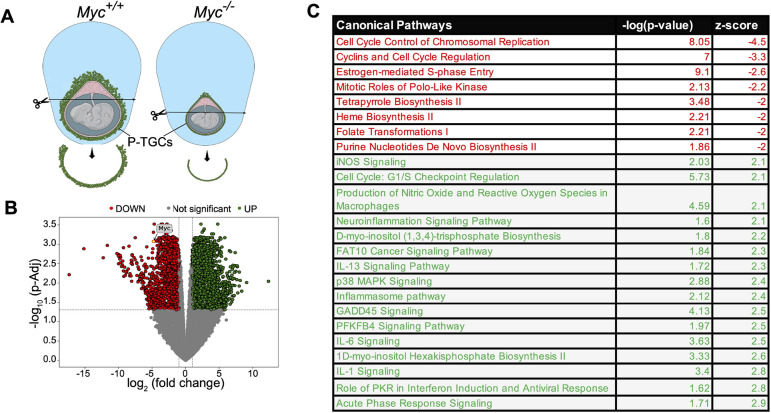
***Myc* promotes normal gene expression in P-TGCs.** (A) Schematic of 9.5 dpc wild-type and *Myc*^−/−^ placenta highlighting the P-TGCs layer used for RNA-seq experiments. (B) A volcano plot for differentially expressed genes (fold change +2.0 and −2.0 and padj-value<0.05) between wild-type and *Myc*^−/−^ P-TGCs. Expression of *Myc* is highlighted in the volcano plot. *n*=4 or 5 biological replicates. (C) Pathway analysis using differentially expressed genes using ingenuity pathway analysis (IPA). Downregulated pathways are highlighted in red, whereas upregulated pathways are highlighted in green (Z score cutoff ±2).

*Myc* encodes a transcriptional regulator that works with RNA polymerase II. One mechanism by which *Myc* could control ploidy is by promoting a gene expression program that allows extra rounds of DNA replication, as has been proposed in *D. melanogaster* (Grendler et al., 2019; Qian et al., 2020; Shcherbata et al., 2004; Zhou et al., 2020). We examined differential gene expression between wild type and *Myc*-null P-TGCs by dissecting the P-TGC layer at the 9.5 dpc stage and performing RNA sequencing (RNA-seq) (Fig. 3A). We collected five independent placentas with wild-type and *Myc* null genotypes. The Spearman correlation for 17,219 expressed genes with hierarchical clustering displayed a good correlation among all five wild-type samples (Fig. S4A). But in *Myc*-null P-TGCs, only four samples correlated well, possibly owing to difficulty in isolation of the thin layer of P-TGCs combined with contamination from other cells. We limited further analysis to five samples from wild type and four from the *Myc* null genotype. To evaluate our TGC isolation, we examined expression of *Prl3d1*, a marker for P-TGCs, and detected expression in both wild-type and *Myc* null P-TGCs, suggesting good quality isolation of the correct cell type. *Myc* mRNA levels were reduced fivefold in the null compared with wild type, confirming the genotype (Fig. 3B).

To broadly compare gene expression programs, we identified differentially expressed genes with a minimum fold change±2.0 and p_adj_-value<0.05. We observed 1312 genes upregulated and 1038 genes downregulated in the *Myc*-null P-TGCs compared with wild type (Fig. 3B). Pathways related to these genes were identified by ingenuity pathway analysis (IPA). We observed a strong reduction of cell cycle control of chromosomal replication (z score<−4.5) and an increase of inflammatory pathways such as IL1, IL6 and IL13 (z score>2.3) (Fig. 3C) without *Myc*. Within the cell cycle control of the chromosomal replication pathway, we observed decreased levels of *Orc1*, *Orc2*, *Mcm2*, *Mcm3*, *Mcm4*, *Mcm5*, *Mcm6*, *Mcm8*, *Cdt1*, *Cdc6*, *Rpa1*, *Rpa2*, *Pola1*, *Pold1* and *Pole* (Fig. S4B,C). We also observed decreased expression of purine nucleotide *de novo* biosynthesis II pathway genes, such as *Atic*, *Gart*, *Pfas* and *Ppat* (Table S2). These results suggest DNA replication components are limiting in the *Myc*-null P-TGCs, similar to observations in salivary glands in *D. melanogaster* lacking Myc (Qian et al., 2020). Induced pathways are mainly related to inflammation and suggest upregulated NFκB activity in *Myc*-null P-TGCs is responsible for activation of several downstream factors related to inflammation (Fig. S4B,D). Although we cannot distinguish between direct and indirect effects, these data suggest that *Myc* supports gene expression related to DNA replication and represses inflammation.

### Loss of *Myc* is associated with genomic instability and senescence

**Fig. 4.**
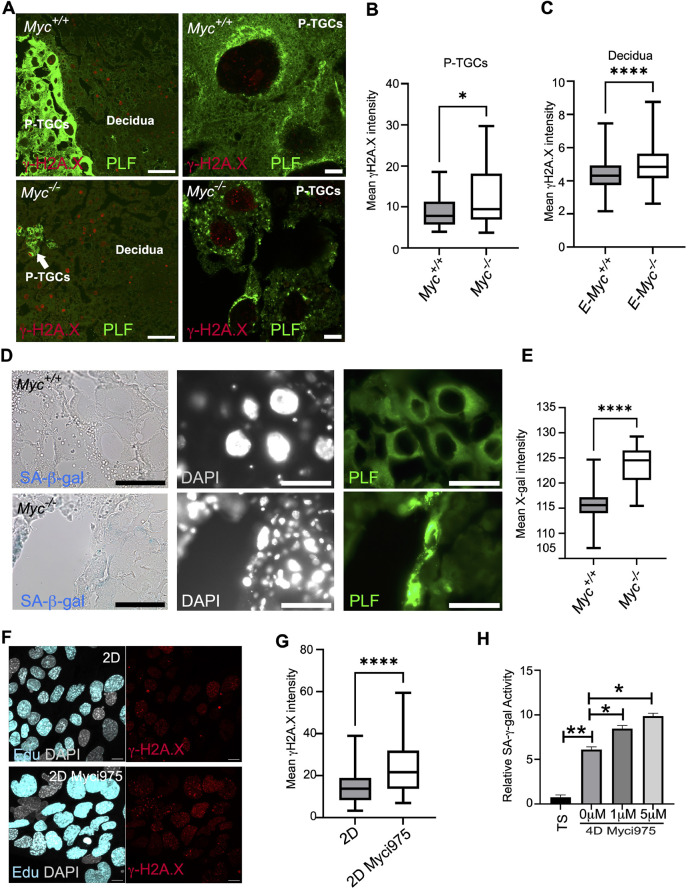
***Myc* prevents DNA damage and senescence in P-TGCs.** (A) 9.5 dpc placental paraffin wax-embedded sections from wild type and *Myc*^−/−^ were immunostained with anti-γH2A.X and anti-proliferin (PLF) antibodies to analyze DNA double-stranded breaks in P-TGCs and decidual cells. P-TGCs are marked with proliferin expression and DAPI was used as a counterstain to mark all nuclei. Scale bars: 100 µm. P-TGCs are highlighted in the zoomed images in the right panel. Scale bars: 10 µm. (B) Box plot showing quantification of mean γH2A.X intensity from P-TGCs of wild-type and *Myc*^−/−^ placenta at 9.5 dpc*. n*=28-31 nuclei from three biological replicates each. *P<0.05. (C) Box plot showing quantification of mean γH2A.X intensity from decidual cells attached to wild-type and *Myc*^−/−^ placenta at 9.5 dpc*. n*=172-193 nuclei from three biological replicates each. ****P<0.0001 (unpaired Student's *t*-test). (D) 9.5 dpc placental cryosections from wild-type and *Myc*^−/−^ were stained for senescence-associated β-gal activity by X-gal staining (blue). P-TGCs were labeled with anti-proliferin antibody and nuclei were counterstained with DAPI. Scale bars: 1 mm. (E) Box plot showing quantification of mean X-gal intensity from P-TGCs of wild-type and *Myc*^−/−^ placenta at 9.5 dpc*. n*=31-35 nuclei from three biological replicates each. ****P<0.0001 (unpaired Student's *t*-test). (F) Two-day differentiated trophoblast stem cells in presence of DMSO or Myci975 were labeled with EdU for 20 h and were analyzed for DNA damage using an anti-γH2A.X antibody. DAPI was used to mark nuclei. Scale bars: 10 µm. (G) Box plot showing quantification of mean γH2A.X intensity in EdU-positive cells from DMSO or Myci975 treatment. *n*=79-98 nuclei from *n*=3 biological replicates. ****P<0.0001 (unpaired Student's *t*-test). (H) Trophoblast stem cells were differentiated for 2 days and DMSO or Myci975 was added for additional 2 days. SA β-gal activity (using ONPG substrate) was measured in trophoblast stem cells and in cells differentiated for 4 days with various treatments. *n*=3 biological replicates. *P<0.05, **P<0.01 (unpaired Student's *t*-test). In box plots, boxes represent interquartile range and whiskers represent minimum and maximum values.

We hypothesized that reduced levels of replication and nucleotide biosynthesis genes may induce replication stress in the *Myc*-null P-TGCs and lead to the accumulation of DNA damage. We immunostained placental sections with γH2A.X, a marker for DNA damage. We observed a significant increase in γH2A.X intensity in the nuclei of *Myc*-null P-TGCs (Fig. 4A,B). We also analyzed DNA damage in the decidual cells associated with either wild-type or *Myc*-null genotypes. Interestingly, decidual cells associated with *Myc*-null P-TGCs showed increased DNA damage (Fig. 4A,C). Because the decidua has at least one copy of *Myc*, this result suggests a non-cell-autonomous induction of DNA damage in the decidual cells. The increase in DNA damage in the *Myc* null P-TGCs is consistent with replication stress caused by insufficient nucleotide biosynthesis and replication factors.

**Fig. 5.**
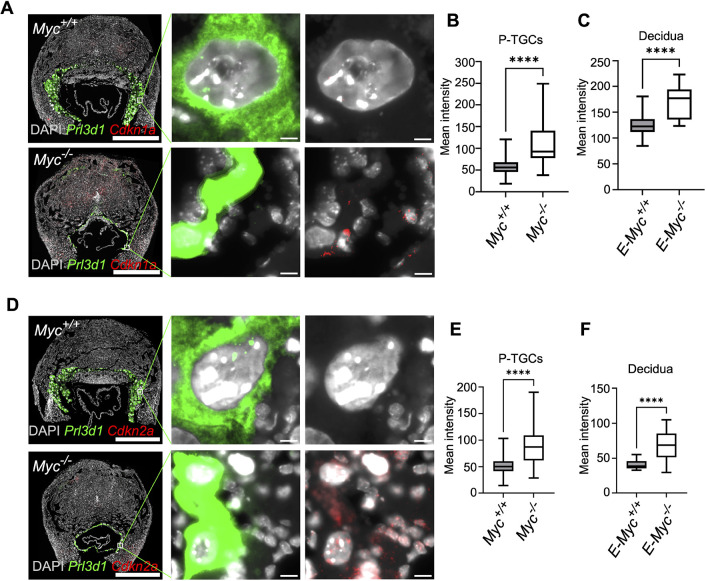
***Myc*^−/−^ P-TGCs show elevation in cell cycle inhibitors by RNA FISH.** (A) RNA-FISH showing expression of *Cdkn1a* in wild-type and *Myc*^−/−^ P-TGCs. P-TGCs are marked with *Prl3d1. n*=3 or 4 biological replicates. Scale bars: 1 mm; 10 µm (enlargements). (B) Box plot showing quantification mean *Cdkn1a* intensity in wild-type and *Myc*^−/−^ P-TGCs. *n*=85-5231 cells. ****P<0.0001 (unpaired Student's *t*-test). (C) Box plot showing quantification of mean *Cdkn1a* intensity in the decidual cells attached to wild-type and *Myc*^−/−^ P-TGCs. *n*=13-31 sections. ****P<0.0001 (unpaired Student's *t*-test). (D) RNA-FISH showing expression of *Cdkn2a* in wild-type and *Myc*^−/−^ P-TGCs. P-TGCs are marked with *Prl3d1. n*=3 or 4 biological replicates. Scale bars: 1 mm; 10 µm (enlargements). (E) Box plot showing quantification mean *Cdkn2a* intensity in wild-type and *Myc*^−/−^ P-TGCs. *n*=150-3644 cells. ****P<0.0001 (unpaired Student's *t*-test). (F) Box plot showing quantification of mean *Cdkn2a* intensity in the decidual cells attached to wild-type and *Myc*^−/−^ P-TGCs. *n*=14-18 sections. ****P<0.0001 (unpaired Student's *t*-test). In box plots, boxes represent interquartile range and whiskers represent minimum and maximum values.

Persistent DNA damage can induce senescence in the placenta (Perez-Garcia and Turco, 2020; Singh et al., 2020). The increase in DNA damage coupled with increased expression of inflammation-associated genes in the *Myc*-null P-TGCs prompted us to analyze senescence by measuring senescence-associated β-gal (SA β-gal) activity in the wild-type and *Myc*-null P-TGCs. We performed SA β-gal staining on placental cryosections at the 9.5 dpc stage, marking P-TGCs with proliferin. We observed SA β-gal staining in *Myc* null P-TGCs but not in wild-type P-TGCs (Fig. 4D,E). We also observed SA β-gal staining in decidual cells associated specifically with the *Myc*-null genotype (Fig. 4D), similar to the DNA damage results. We measured expression of other senescence markers, such as *Cdkn1a* and *Cdkn2a*, in P-TGCs and decidual cells using RNAscope. Consistent with SA β-gal staining, we observed an increase in *Cdkn1a* and *Cdkn2a* mean intensity in the *Myc*-null P-TGCs when compared with wild-type P-TGCs (Fig. 5A,B,D,E). *Cdkn1a* and *Cdkn2a* expression was also increased in decidual cells adjacent to *Myc* null placenta (Fig. 5A,C,D,F). These results suggest *Myc*-null P-TGCs and associated decidual cells display signatures of genomic instability and senescence.

To further examine the cell autonomous role of *Myc* in DNA damage and senescence, we used a trophoblast stem cell culture model (Singh and Gerton, 2021) and the small molecule Myci975 to inhibit MYC function (Han et al., 2019). Myci975 binds to MYC and disrupts the MYC-MAX interaction, targeting MYC for proteasomal degradation (Han et al., 2019). We tested different doses of the Myci975 inhibitor in trophoblast stem cells. We observed significantly lower levels of MYC protein levels at a concentration of 5 µM (Fig. S5A,B). Next, we measured expression of MYC protein at different time points of differentiation. Trophoblast stem cells showed the maximum expression of MYC protein (Fig. S5C,D). The level of MYC protein decreased on day 4 of differentiation. At day 8 of differentiation, the level of MYC protein was minimal. We further analyzed levels of MYC with differentiation and dose of Myci975. After 4 days of differentiation in the presence of 1 µM Myci975, there was no significant reduction in MYC protein levels. In contrast, after 4 days of differentiation in the presence of 5 µM Myci975, a significant reduction in MYC protein levels was observed (Fig. S5E,F). After 8 days of differentiation, cells cultured with 1 µM or 5 µM Myci975 showed a trend of lower MYC. These results suggest that 1 µM and 5 µM Myci975 can inhibit MYC in a dose-dependent manner.

We measured the effect of Myci975 on cell ploidy using flow cytometry. Cells differentiated for 4 days in the presence of 5 µM Myci975 had a decreased >6N population (Fig. S5G,H), suggesting reduced ploidy, mirroring our observations *in vivo* in *Myc*-null mice. Next, we measured DNA damage using γH2A.X staining after 2 days of differentiation in the presence of Myci975. Because MYC regulates replication genes, we measured DNA damage in replicating cells by labelling with EdU. Our results show an increase in DNA damage in the replicating cells in the presence of Myci975 (Fig. 4F,G). To understand the role of DNA damage in senescence *in vitro*, we measured SA β-gal using ONPG (ortho-nitrophenyl-β-galactoside) as a chromogenic substrate (Singh et al., 2020). Consistent with a previous report (Singh et al., 2020), trophoblast stem cells showed minimal senescence but senescence significantly increased after differentiation (Fig. 4H). Addition of Myci975 increased senescence in a dose-dependent manner (Fig. 4H). These results suggest that MYC protects trophoblast cells from DNA damage and senescence.

### *Myc* regulates rRNA transcription in placental cells

**Fig. 6.**
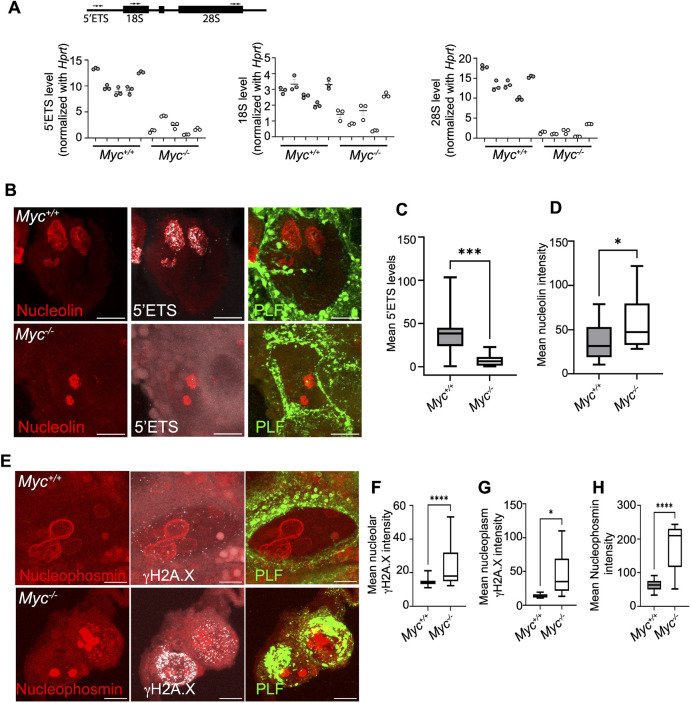
***Myc* promotes nascent rRNA transcription in P-TGCs.** (A) qRT-PCR for 47S rRNA transcription from wild-type and *Myc*^−/−^ P-TGCs. Primer pairs used to measure nascent 47S rRNA (5′ETS) and mature rRNA (18S and 28S) are highlighted. *Hprt* was used to normalize gene expression. *n*=5 biological replicates each. (B) Nascent 47S rRNA (5′ETS) transcription from wild-type and *Myc*^−/−^ P-TGCs. Cryosections were labeled with 47S rRNA 5′ETS probe to detect nascent rRNA transcription; anti-nucleolin was used to mark the nucleolus; anti-proliferin antibodies were used to mark P-TGCs. Scale bars: 10 µm. (C) Box plot showing quantification of mean 5′ETS intensity from P-TGCs of wild-type and *Myc*^−/−^ placenta at 9.5 dpc*. n*=9-31 nuclei from three biological replicates each. ***P<0.001 (unpaired Student's *t*-test). (D) Box plot showing quantification of mean nucleolin intensity from P-TGCs of wild-type and *Myc*^−/−^ placenta at 9.5 *dpc. n*=9-31 nuclei from three biological replicates each. *P<0.05 (unpaired Student's *t*-test). (E) DNA damage in wild-type and *Myc*^−/−^ P-TGCs nucleoli. Paraffin wax-embedded sections were labeled with anti-γH2A.X antibodies to mark DNA damage; anti-nucleophosmin antibodies were used to mark the nucleolus; anti-proliferin antibodies were used to mark P-TGCs. Scale bars: 10 µm. (F) Box plot showing quantification of nucleolar mean γH2A.X intensity from P-TGCs of wild-type and *Myc*^−/−^ placenta at 9.5 dpc*. n*=29-37 nuclei. ****P<0.0001 (unpaired Student's *t*-test). (G) Box plot showing quantification of nucleoplasmic mean γH2A.X intensity from P-TGCs of wild-type and *Myc*^−/−^ placenta at 9.5 dpc*.* Nucleoplasmic mean γH2A.X intensity was calculated by subtracting total nucleolar mean intensity from the nuclear mean intensity. *n*=29-37 nuclei. *P<0.05. (H) Box plot showing quantification of mean nucleophosmin intensity from P-TGCs of wild-type and *Myc*^−/−^ placenta at 9.5 dpc*. n*=29-37 nuclei. ****P<0.0001 (unpaired Student's *t*-test). In box plots, boxes represent interquartile range and whiskers represent minimum and maximum values.

Growth and polyploidy are interdependent and linked with high metabolic activity in endocycling cells (Britton et al., 2002; Zielke et al., 2011). One mechanism by which *Myc* could support high metabolic activity is its demonstrated ability to bind to and regulate transcription of rRNA genes by RNA polymerase I and ribosome biogenesis (Grandori et al., 2005; Grewal et al., 2005; Potapova et al., 2019). To understand how rRNA transcription is affected by loss of *Myc* in P-TGCs, we measured nascent rRNA transcripts. First, we dissected wild-type and *Myc* null P-TGCs from 9.5 dpc placenta and performed qPCR to analyze nascent 47S preribosomal RNA (pre-rRNA) along with mature 18S and 28S rRNA. We used primers to detect the 5′ external transcribed spacer (5′ETS) for 47S preribosomal RNA, and for 18S and 28S mature rRNA using qPCR, and normalized to *Hprt* (Singh et al., 2015; Watada et al., 2020) (Fig. 6A). We observed a severe reduction in 47S preribosomal RNA in *Myc*-null P-TGCs when compared with wild-type P-TGCs (Fig. 6A). Next, we measured the level of mature rRNA and observed a significant reduction in 28S rRNA levels in *Myc*-null P-TGCs when compared with wild-type P-TGCs (Fig. 6A). We observed a less significant reduction in 18S rRNA in *Myc*-null P-TGCs when compared with wild-type P-TGCs (Fig. 6A), possibly owing to the stability of mature 18S rRNA compared with 28S rRNA (Venkov and Hadjiolov, 1969). We acknowledge our data do not distinguish between direct and indirect effects, but are consistent with previous studies indicating that *Myc* acts as a transcription factor for RNA Pol I.

To extend and further validate the qPCR results, we performed ViewRNA FISH using a probe to the 5′ETS to detect nascent rRNA transcripts in histological sections from 9.5 dpc placenta (Cui and Tseng, 2004; Qian et al., 2006). First, we analyzed rRNA in different placental and decidual cells. We observed high expression of rRNA transcripts in P-TGCs and other placental cells when compared with decidual cells, supporting the idea that P-TGCs are metabolically robust (Fig. S6A,B). Next, we compared nascent rRNA transcripts in wild type and *Myc*-null P-TGCs. Consistent with our qPCR results, we observed a significant reduction in nascent rRNA transcripts in *Myc*-null P-TGCs (Fig. 6B,C). Furthermore, nucleoli are shrunken and condensed compared with wild type, suggesting reduced rRNA transcription in nucleoli (Fig. 6B,D) and consistent with nucleolar stress (Potapova et al., 2022 preprint; Yang et al., 2018).

To evaluate whether rDNA was the main source of DNA damage in P-TGCs lacking *Myc*, we measured levels of γH2A.X within nucleoli, using nucleophosmin as a marker. The shrunken nucleoli appear to concentrate nucleophosmin as its intensity is increased in *Myc*-null P-TGCs (Fig. 6H). We observed an increase in DNA damage in the nucleoli of *Myc*-null P-TGCs when compared with wild type (>1.7 fold) (Fig. 6E,F). However, the increase in DNA damage was similar between nucleoli and nucleoplasm, suggesting rDNA is not the only site of damage (Fig. 6F,G).

The reduced levels of transcription without *Myc*, by RNA pol I at the rDNA and by RNA pol II in replication and purine biosynthesis genes, could diminish transcription coupled repair and allow DNA damage to accumulate (Hanawalt and Spivak, 2008). To test this idea, we inhibited transcription in culture using differentiated trophoblast stem cells and measured damage. We used small molecules BMH-21 (Jacobs et al., 2022) and actinomycin D (Sobell, 1985) to inhibit RNA Pol I transcription for 20 h and quantified DNA damage in both nucleoli and nucleoplasm. Treatment with BMH21 causes the expected nucleolar stress phenotype in which nucleoli shrink and round up (Potapova et al., 2022 preprint), reminiscent of the nucleoli in *Myc*-null P-TGCs (Fig. S6C). Interestingly, DNA damage was unchanged in nucleoli and nucleoplasm after BMH21 treatment (Fig. S6C,D), suggesting that low RNA pol I activity cannot account for the increased damage. Consistent with the literature, we observed an increase in overall DNA damage after actinomycin D treatment (Fig. S6C,E). However, actinomycin D treatment causes diffusion of nucleolar proteins, and we were unable to compare DNA damage in nucleoli versus nucleoplasm. These results suggest that reduced transcription does not necessarily lead to increased DNA damage. Our data are most consistent with the increase in DNA damage upon loss of *Myc* stemming from deficiency in nucleotide biosynthesis and DNA replication machinery, due to reduced gene expression. Overall, we conclude that MYC, in addition to supporting transcription by RNA pol II, is also required for rRNA expression in P-TGCs. We speculate that robust rRNA production is required for ribosome biogenesis to support multiple rounds of DNA replication and general metabolic robustness in P-TGCs.

## DISCUSSION

We provide evidence that many mature mouse placental cell types are polyploid. We demonstrate that MYC supports expression of replication and nucleotide biosynthesis genes as well as high levels of rRNA production in P-TGCs. We speculate that, together, these replication and transcription programs are required to create robust highly metabolically active cells. In the absence of *Myc*, P-TGCs accumulate DNA damage and senesce. Previous work has demonstrated that *Myc* is crucial for the development of the placenta and blood lineages in a developing embryo (Dubois et al., 2008). Our study highlights the importance of *Myc* in the development of P-TGCs to support embryo health and survival.

Programmed tissue-specific polyploidy is common throughout evolution, suggesting a conserved evolutionary benefit. Somatic polyploidy is often associated with tissue boundaries and robustness to environmental stress (Lee et al., 2009). One mechanism for achieving polyploidy is endocycling, a modified cell cycle that requires a common program of altered activity of M-phase cyclins, as shown in *D. melanogaster* salivary glands, follicle cells*,* fat body, *A. thaliana* pavement cells and *M. musculus* TGCs (Edgar et al., 2014). Myc and TOR are known to be crucial regulators of endocycles in *D. melanogaster*, but molecular regulators in other species and cell types are unknown. We suggest that P-TGCs are generated via endocycles, based on the loose clusters of DNA-FISH signal in a single nucleus, but further work will be required to establish the mechanism by which polyploidy is achieved in each placental cell type. In *D. melanogaster*, Myc is not required for cell division, but is essential for endocycling, cell size and expression of DNA replication factors (Maines et al., 2004; Pierce et al., 2004). We show that, in mouse, *Myc*-null placenta reaches mid-gestation, suggesting *Myc* is not essential for cell division, but the number and polyploidy of P-TGCs is drastically reduced, suggesting a role for *Myc* in promoting proliferation and polyploidy. An embryo-specific *Myc* knockout generates embryos that are morphologically normal up to 10.5 dpc with no apparent phenotype (Dubois et al., 2008), suggesting the role of *Myc* in proliferation and polyploidy regulation is placenta specific. Consistent with *Myc* regulating ploidy, *Myc* is essential for polyploidy in megakaryocytes but not for cytoplasmic maturation (Guo et al., 2009). We further demonstrate that transcriptional and DNA replication processes are severely compromised without *Myc*, and senescence ensues, providing a significant advance regarding the cellular processes supported by *Myc* and the adverse outcomes in its absence. Altogether, our work suggests that *Myc* is a common and evolutionarily conserved component of a cellular program that licenses polyploidy. Given the conservation of the program between flies and mice, we speculate that human placental polyploid cells, extra villous trophoblasts, may use similar factors to achieve polyploidy, including *MYC*.

Before our study, trophoblast giant cells and syncytiotrophoblasts were recognized as the major polyploid cell types in the placenta (Gerbaud and Pidoux, 2015; Simpson et al., 1992; Soygur and Sati, 2016; Ullah et al., 2008, 2009a,b; Zybina and Zybina, 2020). Our study provides evidence at the chromatid level that multiple additional placental cell types are polyploid, including sinusoidal trophoblast giant cells, spongiotrophoblasts, glycogen cells and the maternal decidua. Our work suggests more exploration of polyploidy in the placenta, and beyond, is warranted, and will be aided by new *in vivo* techniques (Matsumoto et al., 2020). In addition, the 5S FISH probe suggests sister copies are loosely associated in these cell types, which suggests polyploidy is generated by an endocycling mechanism, a finding that bears further investigation. One advantage of multiple rounds of DNA replication is the potential to control gene dose by over- and under-replication of specific regions. For example, in P-TGCs, some genes needed in high amounts are highly amplified to provide a plethora of copies for transcription (Hannibal and Baker, 2016). It is unknown whether other polyploid placental cells are perfect polyploids, but uneven DNA replication would constitute a mechanism to create a customized genome to aid in specifying cell identity.

In addition to *Myc*, our sc-RNAseq results highlighted two additional features of polyploid placental cells relative to diploid cells: (1) suppression of inflammatory pathways; and (2) activation of mTOR1. The recognition of these two features can be broadly rationalized in the context of polyploidy and placental function. First, the suppression of inflammatory pathways in the polyploid cells of the placenta may aid in the protection of the fetus from the immune response of the mother, as half the genetic material is derived from the father and could be recognized as ‘foreign’. Although polyploid placental cells showed high mTOR1 activity, our single cell data suggests that decidual cells have low mTOR1 activity. Interestingly, activation of mTOR1 in the decidua can have negative consequences on birth timing (Hirota et al., 2011), but high translational activity in placental cells could be driven by both mTOR and *Myc* (Saxton and Sabatini, 2017). Along with *Myc*, mTOR is an activator of polyploidy in *D. melanogaster* polyploid cells (Edgar et al., 2014). This suggests mTOR is another evolutionarily conserved factor for the polyploid program and is consistent with high translational activity as a general feature in polyploid cells. A better understanding of the molecular details of these signatures will require further investigation but will enhance our view of how polyploidy is accomplished and its advantages in different contexts.

The decidua is in direct contact with embryo-derived placental tissue but is derived from maternal tissue, and therefore contains the maternal genotype. In our studies we found, surprisingly, that polyploidy is also a feature of normal decidual cells. These cells have lower levels of nascent rRNA, suggesting they are not as translationally active as adjacent P-TGCs. In *Myc*-null embryos and placentas, the maternal genotype still includes at least one copy of *Myc* and the decidua appears mostly normal by histology. Moreover, polyploidy is retained, demonstrating independence from the ploidy of the adjacent P-TGCs. However, decidual cells in this context display elevated DNA damage and senescence, suggesting that a healthy decidua depends on a well-developed functional placenta.

Fetal growth restriction occurs in ∼5% of human pregnancies and preterm birth occurs in ∼10% of human pregnancies in the USA (Blencowe et al., 2012). In tribal populations of India and Sub-Saharan Africa, ∼20% of pregnancies exhibit fetal growth restriction and preterm birth (Kumari et al., 2021; Shah et al., 2021; Walani, 2020). Both conditions are associated with smoking and alcohol consumption. Studies have shown that cigarette smoke and alcohol can damage DNA. Previous reports have suggested that placental senescence is a normal signal that triggers parturition and birth (Hirota et al., 2011). Our study suggests that genotoxic stress in the placenta, created by the loss of *Myc*, hinders placental development and embryo viability, and is associated with DNA damage and premature senescence in the placenta. Extrapolating to a human pregnancy, we speculate that environmental genotoxins, such as cigarette smoke or alcohol, could hamper placental development, trigger premature placental senescence, and lead to fetal growth restriction and/or preterm birth.

In conclusion, we provide evidence that *Myc* is an essential regulator of cell ploidy and senescence in the placenta. It regulates nucleotide biosynthesis and replication genes in the placental cells, along with rRNA transcription; this is a conserved role for *Myc* in polyploid cells from *Drosophila* to mammals. Future studies will no doubt identify more cases of naturally occurring polyploidy, providing more opportunity to determine whether *Myc* is a universal regulator. Furthermore, given that *Myc* is an oncogene, and polyploidy is a hallmark of cancer, it will be interesting to explore whether one oncogenic function of *Myc* is driving polyploidy to create translationally robust stress-resistant cancer cells.

## MATERIALS AND METHODS

### Cell culture

Trophoblast stem cells, derived in the lab, were cultured as described previously (Singh and Gerton, 2021; Singh et al., 2020). For inhibiting *Myc* function, MYCi975 (MedChem Express, HY-129601) was added at different doses after dilution in DMSO for the time indicated. For labelling replicating cells, 1 µM EdU was added for 20 h. For inhibiting RNA Pol I activity, 0.5 µM BMH21 (Sigma-Aldrich, SML1183) or 0.1 mg/ml actinomycin D (Alfa Aesar, J60148) was added for 20 h. Cells were fixed in 4% paraformaldehyde (PFA) for 10 min at room temperature and immunostaining was performed.

### Mouse breeding

All animal experimental protocols were approved by the Institutional Animal Care and Use Committee of the Stowers Institute for Medical Research and were performed according to the protocol. *Myc* floxed (fl/fl) mice were ordered from Jackson Laboratories (MMRRC,32046-JAX) (de Alboran et al., 2001). For the F1 generation, *Myc* floxed (fl/fl) female mice were bred with *Zp3*-Cre male mice (de Vries et al., 2000). For the F2 generation, *Myc* fl/+:*Zp3*-Cre females were crossed with wild-type C57Bl6/J males to generate *Myc*^+/−^ males. In the F3 generation, *Myc*^+/−^ males and *Myc* fl/+:*Zp3*-Cre or *Myc*^+/−^ females were crossed to generate *Myc*^+/+^, *Myc*^+/−^ and *Myc*^−/−^ embryos. Tail snips, embryos or yolk sacs were used for genotyping.

### Histology

For paraffin wax-embedded sections, placentas at 9.5 or 14.5 dpc were harvested in PBS and fixed in 4% PFA (Ted Pella, 18505) at 4°C overnight. After fixation, placentas were dehydrated and embedded in paraffin wax blocks. Embedded placentas were sectioned at 5 or 10 µm and mounted on positively charged slides (Thermo Fisher Scientific, 12-550-15). For cryosections, placentas at 9.5 dpc were harvested in PBS and fixed in 1× fixative provided in the β-galactosidase staining kit (Cell Signaling Technology, 9860) for 30 min at room temperature. Fixed tissues were washed three times with PBS and kept overnight in 30% sucrose/PBS solution at 4°C. The next day, tissues were embedded in OCT compound (Tissue Tek, 4583) and 16 µm sections were collected and mounted on positively charged slides. Slides were stored at −80°C until further analysis.

### Immunofluorescence and immunohistochemistry

Trophoblast stem cells and differentiated cells were cultured in 30 mm dishes (MatTek, P35G-0-14-C) and fixed in 4% PFA for 10 min at room temperature. Cells were washed three times with PBS. For measuring replicating cells, the Click-iT EdU cell proliferation kit (Thermo Fisher Scientific, C10340) was used according to the manufacturer's instructions. Briefly, cells were permeabilized with 0.5% Triton-X 100 (J. T. Baker, X200-07) in PBS for 20 min at room temperature. Cells were washed with PBS containing 3% BSA (Thermo Fisher Scientific, 15260037). Click-iT reaction cocktail was prepared as per kit instructions and applied to the cells. The reaction was carried out for 60 min at room temperature. The reaction cocktail was removed and cells were incubated with PBS containing 3% BSA. To perform immunostaining, cells were blocked in PBST (0.05% Tween-20, Sigma-Aldrich, P9416) containing 5% donkey serum (Sigma-Aldrich, D9663) for 1-2 h. Primary antibodies against proliferin (R&D Systems, AF1623) (1:500), nucleolin (Abcam, ab22758) (1:500), nucleophosmin (Santa Cruz Biotechnology, sc-32256) (1:100), MYC (Abcam, ab32072) (1:200) and γH2A.X (Cell Signaling Technology, 9718) (1:200) were diluted in blocking buffer and incubated overnight at 4°C. The next day cells were washed with PBST three times and incubated with secondary antibodies Alexa Fluor-555 (Thermo Fisher Scientific, A21432), Alexa Fluor-488 (Thermo Fisher Scientific, A11055), Alexa Fluor-555 (Thermo Fisher Scientific, A31570), Alexa Fluor-647 (Thermo Fisher Scientific, A31573), Alexa Fluor-647 (Thermo Fisher Scientific, A21447), Alexa Fluor-555 (Thermo Fisher Scientific, A32727) and Alexa Fluor-488 (Thermo Fisher Scientific, A21245) at 1:500 dilution. Cells were washed and mounted in DAPI (Vector Laboratories, H-1200). For immunohistochemistry of paraffin wax-embedded tissue, sections were dewaxed, and hydrated, and antigen retrieval was performed using 0.1 M citrate buffer (pH 6.0) at 95°C for 10 min in the EZ Retriever system V2. Blocking and antibody incubation was performed as mentioned above. For immunohistochemistry of cryosections, tissues were thawed at room temperature and fixed again with 4% PFA for 10 min at room temperature. Sections were washed three times with PBS, blocked and incubated with antibodies, as mentioned above. Sections were imaged using a LSM780 or LSM800 confocal microscope (Zeiss).

### DNA immunoFISH

DNA-FISH was performed as described previously with some modification (Yu and Potapova, 2022). Trophoblast stem cells were grown on 22 mm coverslips and fixed in 4% PFA for 10 min at room temperature. Paraffin wax-embedded sections were dewaxed and hydrated, and antigen retrieval was performed using 0.1 M citrate buffer (pH 6.0) at 95°C for 10 min in the EZ Retriever system V2. Cryosections were thawed and fixed in 4% PFA for an additional 10 min. After fixation, specimens were washed with PBS twice. Specimens were treated with RNAse A (Qiagen, 19101) in PBS (0.1 mg/ml) for 30 min at 37°C. Specimens were washed again with PBS twice and incubated in 25% glycerol/PBS for a minimum of 1 h at room temperature. After incubation, coverslips or slides were dipped in liquid N_2_ for ∼6 s and returned to 25% glycerol/PBS. This process was repeated twice and rinsed with 0.1 N HCl and again incubated in 0.1 N HCl for 5 min. After HCl treatment, cells were washed with PBS twice and once with 2× SSC buffer. Cells were preincubated in 2× SSC/50% deionized formamide (Millipore, S4117) for at least 6 h at 4°C. 2 µl 5S, *45S* and *Prl8a8* probes were diluted [Empire Genomics, RPCI-23-339I8 (*5S*), RPCI-23-225M6 (*45S*) and RPCI-23-117C5 (*Prl8a8*)] in 8 µl hybridization buffer provided with the probe. The probe was denatured for 2 min at 85°C and transferred immediately to ice for 2 min. 10 µl of the denatured probe was applied to the cells, covered with a coverslip and sealed with sealant (Cytobond, 2020-00-1). Specimens were denatured on a heat block for 5 min at 80°C. Sealed specimens were incubated in a moist chamber at 37°C for 24 h. The next day, specimens were washed with washing buffer I (50% formamide/2× SSC) at 45°C for 5 min twice and with washing buffer II (1× SSC) at 45°C for 5 min twice. After washing, specimens were incubated in PBS and prepared for immunostaining. Cells were blocked in PBST (0.05% Tween-20, Sigma-Aldrich, P9416) containing 5% donkey serum (Sigma-Aldrich, D9663) for 1 h. Primary antibodies against proliferin (R&D Systems, AF1623) was diluted (1:200) in blocking buffer and incubated overnight at 4°C. The next morning, cells were washed three times in PBST and incubated for 2 h with secondary antibodies conjugated with Alexa Fluor-555 or 647 (Thermo Fisher Scientific A21432 and A21447; 1:500) in blocking buffer. After secondary antibody incubation, cells were washed three times in PBST. Cells were mounted in DAPI (Vector Laboratories, H-1200). Slides were imaged using a LSM780 or LSM800 confocal microscope (Zeiss) and *z*-stacks were taken.

### scRNA-seq

14.5 dpc placenta was isolated in D-PBS (Thermo Fisher Scientific, 14287072) and the adult brain dissociation kit (Thermo Fisher Scientific, 130-107-677) was used to make a single cell suspension according to the manufacturer's instructions. Each 14.5 dpc placenta was cut into four small pieces using a scalpel blade. Placental pieces were transferred in gentleMACS C tube (Miltenyi Biotec, 130-093-237) and 1950 µl of Enzyme mix 1 (Buffer Z 1900 µl+Enzyme P 50 µl) was added. A further 30 µl of Enzyme mix 2 (Buffer Y 20 µl+Enzyme A 10 µl) was added to the same tube. The 37C_ABDK_01 program of gentleMACS was used and once the program was completed, cells were centrifuged at 300 ***g*** for 5 min. The pellet was resuspended in ice-cold 10 ml D-PBS and filtered through a 70 µm MACS SmartStrainer (Miltenyi Biotec, 130-098-462) using a 50 ml falcon tube. The C tube was washed again with 10 ml ice-cold D-PBS and filtered with the same SmartStrainer. The MACS SmartStrainer was discarded and the cell suspension was centrifuged at 300 ***g*** for 10 min at 4°C. The supernatant was removed and the pellet was resuspended in ice-cold 1550 µl D-PBS and transferred to a 15 ml falcon tube. Debris removal solution was added (450 µl) and mixed well. Ice-cold D-PBS (2 ml) was gently overlaid, and the tube was centrifuged at 4°C and 3000 ***g*** for 10 min. Three phases were formed. The top two layers were discarded, and tube was filled with 10 ml ice-cold D-PBS. The tube was gently mixed and centrifuged at 4°C and 1000 ***g*** for 10 min. The pellet was resuspended in 0.5 ml of 1× red blood cell removal solution and incubated for 10 min at 4°C. Ice-cold D-PBS (5 ml) was added and centrifuged at 4°C and 300 ***g*** for 10 min. The pellet was resuspended in 0.5 ml ice-cold PBS and the acridine orange (AO)/propidium iodide (PI) method was used to quantify cell viability. Samples with viability above 87% were used for further sequencing. The Chromium Next GEM Single Cell 3′ Reagent Kit v3.1 was used to make the cDNA library using 10X Genomic reagents (Next GEM Chip G Single Cell Kit 1000120, Next GEM Single Cell 3′ Gel Bead Kit v3.1 1000122, Single Cell 3′ Gel Beads v3.1 2000164, Next GEM Single Cell 3′ GEM Kit v3.1 1000123, Next GEM Single Cell 3′ Library Kit v3.1 1000157, i7 Multiplex Kit 120262, DynaBead – MyOne Silane Beads 2000048 and SPRIselect Beads 17201600). Sequencing was performed using Illumina Nova-Seq-SP. Raw reads were demultiplexed and aligned to mm10 reference genome from UCSC using the 10X Genomics CellRanger pipeline (v 3.0.0) with gene model retrieved from Ensembl, release 102. Downstream analysis was performed in R (v 4.1.2) with Seurat package (v 4.1.0). Samples were merged using Seurat merge and cells with fewer than 200 genes expressed or greater than 8000 genes expressed, as well as cells with mitochondrial expression greater than 10% were removed from the data. Data were normalized using Seurat sctransform and the first 70 principal components were used for downstream steps. For identifying RBC clusters, we used a resolution of 1 to obtain many clusters with small cell populations. Cluster markers were identified using Seurat FindAllMarkers with the minimum fraction of cells in either of the clusters expressing the gene, set to 0.25 and a log fold change threshold of greater than or equal to 0.25. The clusters that had positive Hba (Hba-x, Hba-a1 and Hba-a2) and Hbb (Hbb-bt, Hbb-bs, Hbb-bh3, Hbb-bh2, Hbb-bh1, Hbb-bh0 and Hbb-y) markers were removed from the analysis. The remaining cells were normalized again, re-clustered with resolution of 0.7 and visualized using UMAP. Cluster markers were identified using Seurat FindAllMarkers with the same parameters mentioned above. Gene expression markers between two different cell types were identified using Seurat FindMarkers with default parameters. Sequencing data have deposited in GEO under accession number GSE215382.

### RNA-seq and qRT-PCR

P-TGCs from 9.5 dpc stage were dissected as mentioned previously (Hannibal and Baker, 2016) in ice-cold PBS and the corresponding embryos were used for genotyping. Total RNA from P-TGCs was isolated using TRIzol (15596026, Thermo Fisher Scientific). rRNA was depleted using the NEBNext rRNA Depletion Kit (Human/Mouse/Rat) (NEB E6310L). cDNA synthesis was performed using the NEBNext Ultra II Directional RNA Library Prep Kit (NEB, E7760S) and NEBNext Multiplex Oligos for Illumina (Index Primer Set 1) (NEB, E7335S). Sequencing was performed using Illumina NextSeq-HighOutput (NextSeq-HO-75). RNA-seq reads were demultiplexed into Fastq format allowing up to one mismatch using Illumina bcl-convert (v 3.10.5) and aligned to mm10 reference genome from the University of California at Santa Cruz (UCSC) using STAR (v 2.7.3a) with gene models retrieved from Ensembl, release 102 to generate gene read counts. The transcript abundance ‘TPM’ (transcripts per million) was quantified using RSEM version 1.3. Differentially expressed genes were determined using the R package edgeR (v 3.34.0) after filtering low-expressed genes with a ‘CPM’ (counts per million) of 0.5 in at least one library. The resulting *P*-values were adjusted with the Benjamini–Hochberg method using R function p.adjust. Genes with an adjusted *P*<0.01 and a fold change of two were considered differentially expressed. We analyzed metabolic pathways and signaling pathways (‘cell cycle regulation’, ‘cytokine signaling’ and ‘cellular immune response’) using customized IPA analysis.

For qRT PCR analysis, 100 ng of total RNA was treated with RNase free DNase to remove any genomic DNA contamination, as per the manufacturer's instructions (Promega, M6101). cDNA synthesis was performed using the first-strand synthesis system (Thermo Fisher Scientific, 18080051). cDNA was amplified using specific primers (Integrated DNA Technologies) and Fast SYBR green master mix (Thermo Fisher Scientific, 4385612) using a QuantStudio 7 Flex Real time PCR machine (Applied Biosystems). Each reaction was performed in triplicate and fold change was calculated using 2^−ΔΔCT^ (Livak and Schmittgen, 2001). Primers used for qPCR (Singh et al., 2015; Watada et al., 2020) are as follows: m_47S_FP, 5′CTCTTAGATCGATGTGGTGCTC3′; m_47S_RP, 5′GCCCGCTGGCAGAACGAGAAG3′; m_18S-FP, 5′GCTTAATTTGACTCAACACGGGA3′; m_18S-RP, 5′AGCTATCAATCTGTCAATCCTGTC3′; m_28S_FP, 5′TGGGTTTTAAGCAGGAGGTG3′; m_28S_RP, 5′GTGAATTCTGCTTCACAATG3′; m_*Hprt*_FP, 5′CCTAAGATGAGCGCAAGTTGAA3′; and m_*Hprt*_RP, 5′CCACAGG ACTAGAACACCTGCTAA3′.

### RNA-FISH

RNA *in situ* experiments were performed using a RNAscope 2.5HD-duplex detection kit (Advanced Cell Diagnostics, 322500). Probes for *Prl3d1* [Mm-Prl3d1-C2 (405521-C2)], *Cdkn1a* [Mm-Cdkn1a (408551)] and *Cdkn2a* [Mm-Cdkn2a (411011)] were purchased from Advanced Cell Diagnostics. For fluorescence detection, RNAscope multiplex fluorescent detection regents V2 (Advanced Cell Diagnostics, 323110), TSATM Plus Cyanine 3 System (PerkinElmer, 2268306) and TSATM Plus Cyanine 5 System (PerkinElmer, 2268813) were used according to the manufacturer's instructions. Slides were scanned using an Olympus VS120 with a 20× objective.

### Nascent-rRNA

Cryo-sections on slides were fixed in 4% PFA for 15 min, washed in PBS three times and dehydrated in 100% ethanol. A hydrophobic barrier was created around the sections on the slide using an ImmEdge Hydrophobic Barrier PAP Pen (Vector Laboratories) and sections were permeabilized with PBS-0.1% Triton X-100 for 20 min. Probes (rRNA ETS) were applied on sections, and RNA FISH was performed with the ViewRNA ISH Cell Assay Kit (Thermo Fisher Scientific, QVC0001) according to the manufacturer's instructions with some modifications (Falcon et al., 2022). Briefly, hybridization was performed in a humid chamber for 16 h at 40°C, sections were washed twice with viewRNA wash buffer for 3 min, twice with 0.5× SSC-0.1% TritonX-100 for 5 min and twice with viewRNA wash buffer for 3 min. Sections were incubated in pre-amplifier solution for 1 h at 40°C, washed twice with viewRNA wash buffer, twice with 0.5× SSC-0.1% TritonX, then twice with viewRNA wash buffer. Incubation in amplifier solution was performed for 1 h at 40°C followed by washing twice with viewRNA wash buffer, twice with 0.5× SSC-0.1% Triton X-100 and twice with viewRNA wash buffer. Label probe mix solution was applied on sections at 40°C for 1 h, then washed with viewRNA wash four times, 0.5× SSC-0.1% Triton X-100 for 10 min and washed with PBS twice. Blocking was performed using superblock (Thermo Fisher Scientific, 37580) for 1 h at room temperature, and sections were incubated with anti-nucleolin antibody (Abcam, ab22758; 1:200) for 16 h at 4°C. After three rinses with PBS, secondary antibody (Invitrogen, #A-31573; 1:500), including 10 µg/ml of DAPI, was applied for 1 h at room temperature and washed with PBS twice. Prolong Gold was applied for mounting media and slides were kept at 4°C until observation.

### SA-β-Galactosidase

Placentas supporting embryos at 9.5 dpc were collected and fixed for 30 min at room temperature in 1× fixative from the senescence β-galactosidase staining kit (Cell Signaling Technology, 9860). Tissues were washed with PBS three times and kept in 30% sucrose overnight at 4°C. The next day, tissues were embedded in OCT compound (Tissue Tek, 4583) and 16 μm sections were collected on positively charged slides. Sections were washed with PBS and staining buffer once each. Finally, sections were stained using a staining solution from the senescence β-galactosidase staining kit for 48 h at 37°C. After staining, sections were washed with PBS three times and immunohistochemistry was carried out using anti-proliferin antibody, as mentioned above. Sections were imaged using an Axioplan 2 microscope (Zeiss). For trophoblast stem cell cultures and differentiated cells, we performed senescence analysis as described previously (Singh et al., 2020).

### Western blot

Cells were lysed in cold protein lysis buffer [10 mM Tris-HCl (pH 7.4), 100 mM NaCl, 1 mM EDTA, 1 mM EGTA, 1% Triton X-100, 10% glycerol, 0.1% SDS, 0.5% sodium deoxycholate, 2 mM Na_3_VO_4_, 10 mM NaF and 1× protease inhibitor cocktail] for 30 min at 4°C and quantified using the micro-BCA kit. Lysate was electrophoresed on a NuPAGE bis-tris 4-12% gradient protein gel (Thermo Fisher Scientific, NP0323BOX) and transferred to a nitrocellulose membrane (Amersham, 10600002). Membranes were blocked in 5% milk using TBST (TBS +0.05% Tween-20) for 1 h and incubated with primary anti-MYC antibody (Cell Signaling Technology, 5605) (1:1000) and anti-Actin antibody (Cell Signaling Technology, 3700, 1:1000) overnight at 4°C. Membranes were washed three times using TBST and incubated in HRP-conjugated secondary antibody (GE Healthcare, NA931V and NA934V). Membranes were washed and developed using the ECL 2 (Thermo Fisher Scientific, 80196) western blotting system. Membranes were scanned using a Typhoon 9400 laser scanner (GE Healthcare).

### Flow cytometry

1×10^5^ trophoblast stem cells (TSCs) were seeded in 100 mm tissue-culture dishes and allowed to attach for 1 day (Singh and Gerton, 2021). After TSCs attached, they were differentiated for 4 days in the presence of either DMSO or 5 µM MYCi975. After 4 days of differentiation, cells were detached using TrypLE and washed with PBS. Cells were fixed in 70% ethanol and stored at −20°C until further analysis. Cells were washed with PBS containing 1% serum and resuspended in 1 ml of citrate buffer (Sigma-Aldrich, S-1282). 3×10^5^ cells were filtered using a 70 µm filter (Filcons, 12 070-67S) and further diluted with 5 ml of citrate buffer. Cells were centrifuged at 500 ***g*** and the concentration was adjusted to 3×10^5^ cells/0.1 ml citrate buffer. 0.1 ml of cells were transferred to a 15 ml falcon tube and 900 µl solution A (Sigma-Aldrich, S-1407) from the DNA staining reagent kit was added and incubated for 10 min at room temperature. 750 µl solution B (Sigma-Aldrich, S-1532) from DNA staining reagent kit was added and incubated for 10 min at room temperature. 750 µl solution C (Sigma-Aldrich, S-1657) from DNA staining reagent kit was added and incubated for 10 min at room temperature. Cells were centrifuged at 500 ***g*** and left in 500 µl of solution. All samples were transferred into 5 ml polypropylene round-bottom tubes (Falcon, 352063) and then run on a five laser ZE5 Cell Analyzer (Bio-Rad). The ploidy of each sample was determined in FlowJo v10.8.1. The proportion of cells for each ploidy group was compared between treated and untreated samples using an Asymptotic Wilcoxon–Mann–Whitney test in R 4.0.2.

### Image analysis

Fiji software was used for quantification (Schneider et al., 2012). Single sections or sum projection was used. Images were converted to 8-bit, except for β-gal quantification, where we converted them to 8-bit color. The background was subtracted, nuclei were thresholded based on DAPI and nucleoli were thresholded based on nucleolin or nucleophosmin. Integrated density, mean, area and foci were counted using Fiji. For RNAscope (Fig. 5), green positive nuclei were found using Cellpose (Stringer et al., 2021). The mean red pixel intensity was quantified and compared with the mean red pixel intensity in the other areas of the tissue. Tissue was identified using the DAPI signal, then blurred and thresholded. GraphPad Prism was used for making graphs, identifying outliers and calculating statistical significance. In all the box plots, boxes represent interquartile range and whiskers represent minimum and maximum values. An unpaired Student's *t*-test was performed between indicated genotypes for statistical analysis.

## Supplementary Material

10.1242/develop.201581_sup1 Supplementary information
