# Supplementary material for: Myc promotes polyploidy in murine trophoblast cells and suppresses senescence
Source: Development. 2023 Jun 6;150(11):dev201581. doi: 10.1242/dev.201581 (PMC10309589; doi:10.1242/dev.201581)
Supplement: Supplementary information [file develop-150-201581-s1.pdf]

Fig. S1

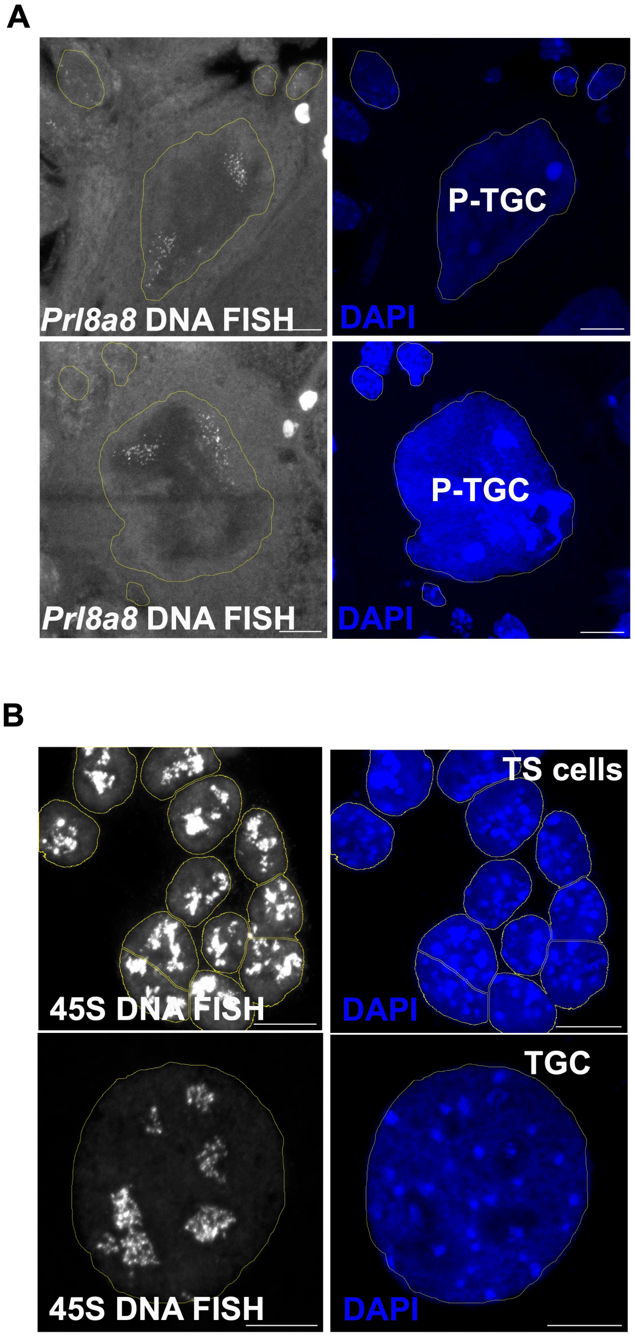

Development • Supplementary information

Fig. S1. DNA fluorescence in situ hybridization to identify ploidy of cells.

(A) DNA FISH using probe spanning *Prl8a8* gene to show the number of copies of the genome in P-TGCs at 14.5 *dpc* using 10 μm paraffin sections. n=3 biological replicates each. These are the maximum projection from z stacks and two P-TGCs are shown as example. (Scale= 10 μm)

(B) DNA FISH using *45S* probe to show the number of copies of the genome in trophoblast stem cells and differentiated TGCs. n= 3 biological replicates each. (Scale= 10 μm)

Fig. S2

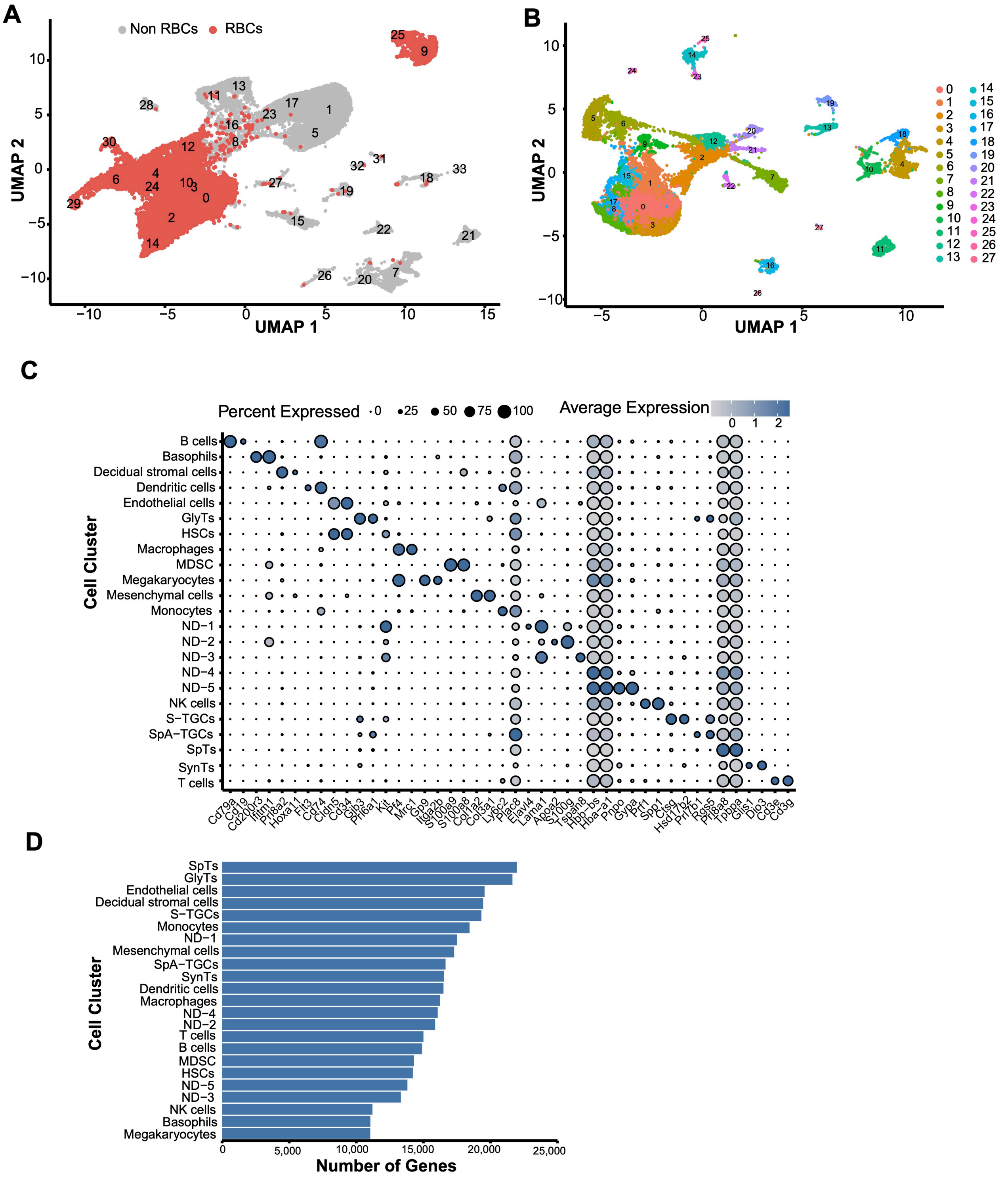

Development • Supplementary information

Fig. S2. Diverse placental cell type identification in mature mouse placenta.

(A) UMAP of 14.5 *dpc* placenta using scRNA seq analysis. Each dot represents a single cell. Cells with hemoglobin gene expression (Hba1 and Hbb) are highlighted in red and marked as red blood cells (RBCs).

(B) After the removal of RBCs, the remaining cells were analyzed and the UMAP illsutrates different cell types.

(C) Based on published markers, each cluster from Fig. S2B is assigned a specific cell type. Five clusters labeled ND1 to ND5 are unassigned.

(D) The bar graph shows the number of genes identified in each specific cell type cluster.

Fig. S3

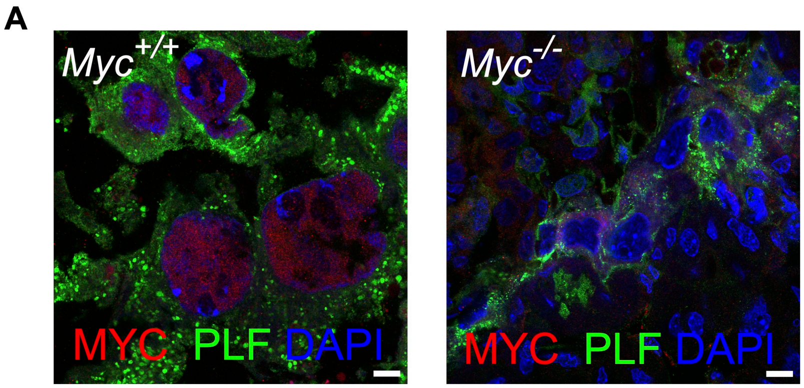

Fig. S3. Oocytes-specific cre (*Zp3-Cre*) is sufficient to knockout floxed *Myc* allele.

(A) Wild type and *Myc*$^{-/-}$ placenta at 9.5 *dpc* were stained with anti MYC and anti-proliferin antibody to analyze MYC expression in P-TGCs. DAPI was used as a counter stain to mark nuclei. n=2 biological replicates each. (Scale= 10 μm)

Development • Supplementary information

Fig. S4

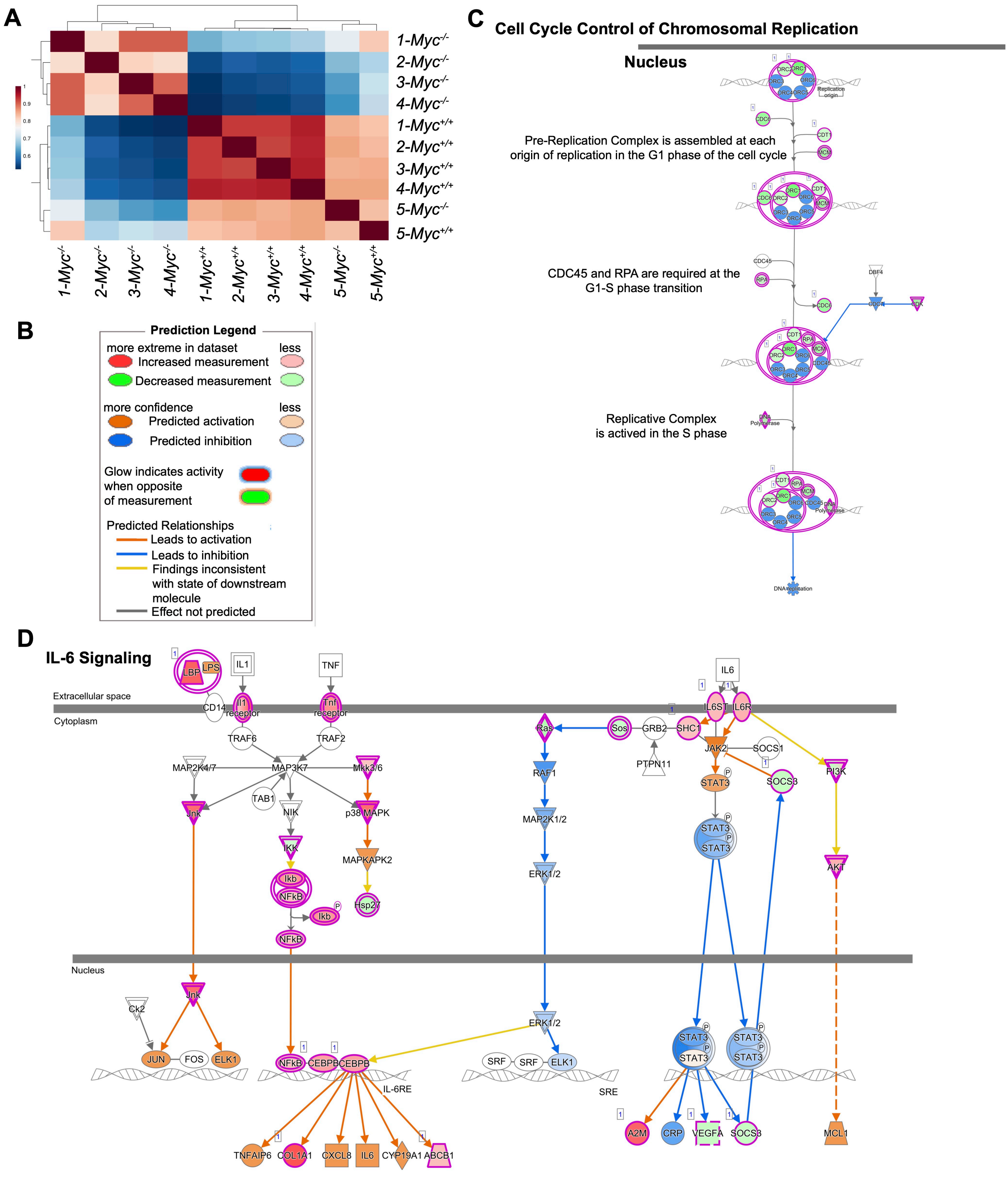

Development • Supplementary information

Fig. S4. Differential gene expression analysis identifies dysregulated pathways in *Myc*$^{-/-}$ TGCs.

(A) Spearman correlation for 17219 expressed genes in P-TGCs with hierarchical clustering.

(B) Canonical pathway activation or repression key in IPA analysis.

(C) Genes in the cell cycle control of chromosomal replication pathway are significantly downregulated in *Myc*$^{-/-}$ TGCs as compared to wild type P-TGCs. Significantly downregulated genes are highlighted in light green including ORC1, ORC2, CDC6, CDT1, RPA, MCM, etc.

(D) Genes in the IL6 signaling pathway are significant upregulated in *Myc*$^{-/-}$ P-TGCs as compared to wild type P-TGCs. Significantly upregulated genes are highlighted in pink such as IL1 receptor, IL6 receptor, Tnf receptor, NFkB, Ikb, CXCl8, IL6, CYP19A1, etc.

Development • Supplementary information

Fig. S5

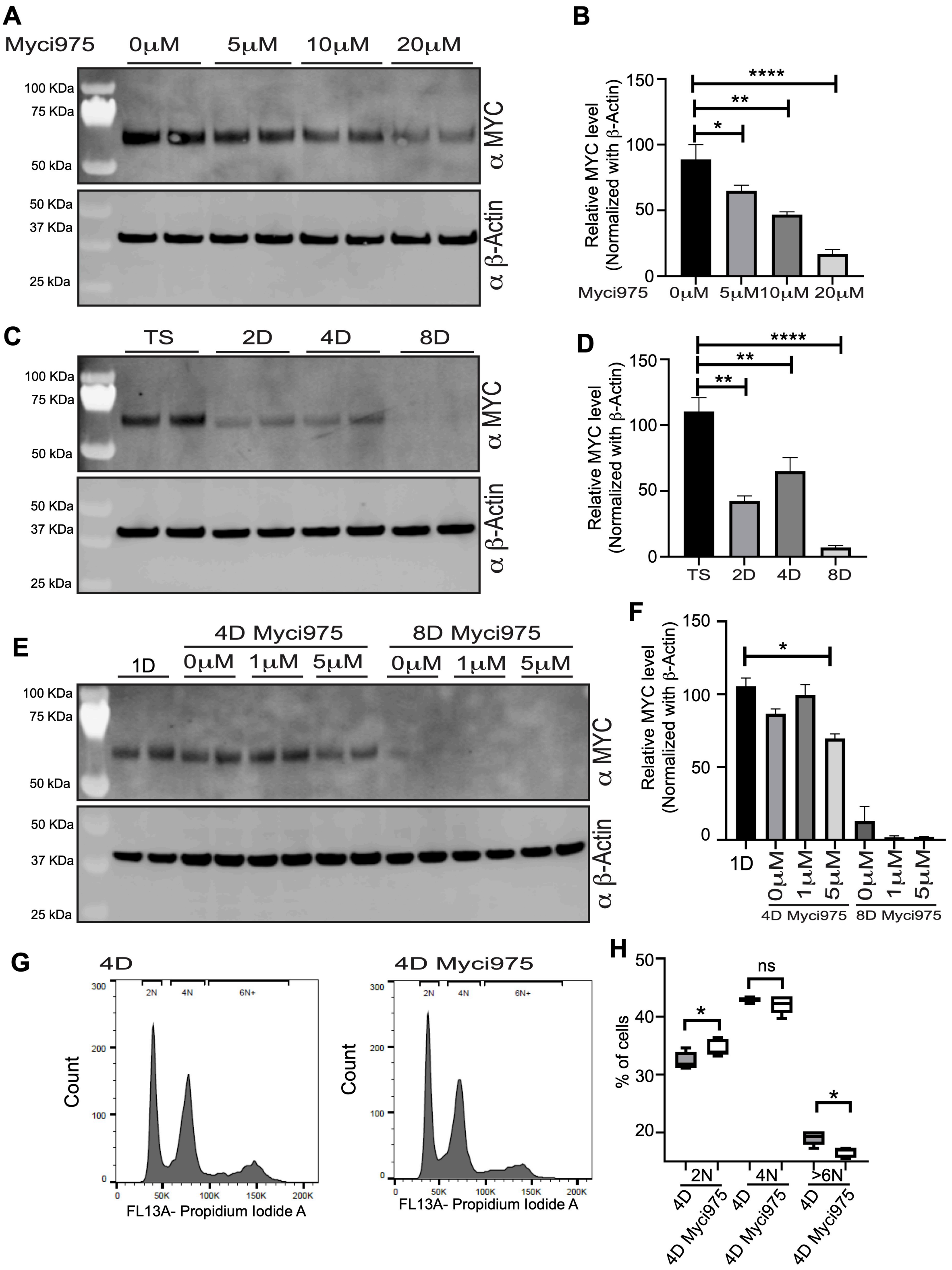

Development • Supplementary information

Fig. S5. *Myc* inhibition in-vitro accelerates senescence and inhibits polyploidy.

(A) Western blot analysis to understand the effect of Myci975 on MYC protein level with indicated doses after 24h of treatment. Anti MYC and anti β-Actin antibody was used to detect MYC and β-Actin proteins respectively.

(B) Quantification of levels of MYC protein relative to β-Actin with indicated doses of Myci975.

(C) Western blot analysis to understand MYC protein levels after differentiation of TS cells with indicated time point. *p<0.05, **p<0.01, and ****p<0.0001.

(D) Quantification of levels of MYC protein relative to β-Actin after differentiation of TS cells.

(E) Western blot analysis to understand MYC protein levels after differentiation with Myci975 with indicated time points and doses. **p<0.01, and ****p<0.0001.

(F) Quantification of levels of MYC protein relative to β-Actin after differentiation of TS cells with different doses of Myci975. *p<0.05.

(G) Ploidy analysis of four days differentiated trophoblast stem cells either in presence of DMSO or Myci975. Y-axis shows count whereas X-axis shows propidium iodide intensity and ploidy.

(H) Quantification of 2N, 4N and >6N cells in presence of DMSO or Myci975. n=5 biological replicates each. *p<0.05.

Development • Supplementary information

Fig. S6

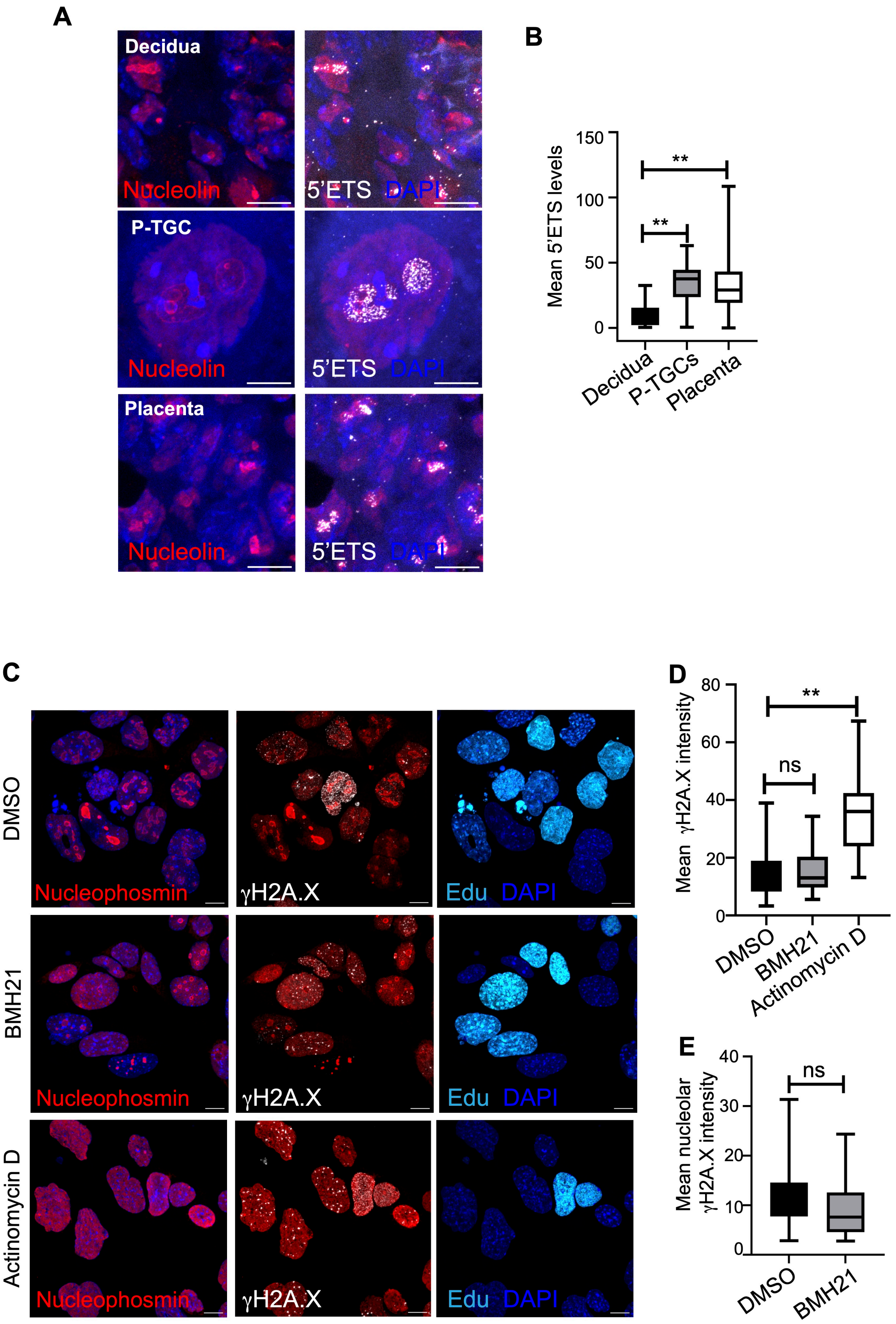
A
Decidua
Nucleolin
5'ETS DAPI
P-TGC
Nucleolin
5'ETS DAPI
Placenta
Nucleolin
5'ETS DAPI
B
Mean 5'ETS levels
150
100
50
0
**
**
Decidua
P-TGCs
Placenta
C
DMSO
BMH21
Actinomycin D
Nucleophosmin
γH2A.X
Edu DAPI
D
Mean γH2A.X intensity
80
60
40
20
0
**
ns
DMSO
BMH21
Actinomycin D
E
Mean nucleolar γH2A.X intensity
40
30
20
10
0
ns
DMSO
BMH21

Fig. S6. Evaluation of rRNA transcription and nucleolar DNA damage in placental cells.

(A) Nascent 47S rRNA (5'ETS) transcription from decidua, P-TGCs and placental cells at 9.5 *dpc*. Cryosections were labeled with 47S rRNA 5'ETS probe to detect nascent rRNA transcription, and anti-nucleolin was used to mark the nucleolus. (Scale= 10 μm)

(B) Box plot showing quantification of mean 5'ETS intensity from decidua, P-TGCs, and other placental cells at 9.5 *dpc.* n=30-69 nuclei from 3 biological replicates each. **$p<0.01$.

(C) Trophoblast stem cells were differentiated for 2 days, then labeled with EdU for 20h in presence of DMSO, BMH21 or Actinomycin D. Cells were analyzed for DNA damage using anti-γH2A.X antibody, and nucleoli were marked with anti-nucleophosmin antibody. DAPI was used to mark nuclei. (Scale= 10 μm)

(D) Box plot showing quantification of mean nuclear γH2A.X intensity in EdU positive cells from. n=76-95 nuclei. **$p<0.01$.

(E) Box plot showing quantification of mean nucleolar γH2A.X intensity in EdU positive cells from. n=76-95 nuclei.

Table S1. Expressed genes in each cluster of Fig. S2A (sheet1) and Fig. S2B (sheet2) from scRNA-seq.

Click here to download Table S1

Table S2. Differential gene expression between wild type and *Myc*$^{-/-}$ P-TGCs.

Click here to download Table S2
