# Supplementary material for: Myc promotes polyploidy in murine trophoblast cells and suppresses senescence
Source: Development. 2023 Jun 6;150(11):dev201581. doi: 10.1242/dev.201581 (PMC10309589; doi:10.1242/dev.201581)
Supplement: Supplementary file 1 [file develop-150-201581_review_history.pdf]

Development

Myc promotes polyploidy in murine trophoblast cells and suppresses senescence

Vijay Pratap Singh, Huzaifa Hassan, Fengyan Deng, Dai Tsuchiya, Sean McKinney, Kevin Ferro and Jennifer L. Gerton

DOI: 10.1242/dev.201581

Editor: Liz Robertson

Review timeline

Original submission:	3 January 2023
Editorial decision:	21 February 2023
First revision received:	17 April 2023
Accepted:	27 April 2023

Original submission

First decision letter

MS ID#: DEVELOP/2023/201581

MS TITLE: c-Myc promotes polyploidy in murine trophoblast cells and suppresses senescence

AUTHORS: Vijay Pratap Singh, Huzaifa Hassan, Fengyan Deng, Dai Tsuchiya, Sean McKinney, Kevin Ferro, and Jennifer Gerton

I have now received all the referees' reports on the above manuscript, and have reached a decision. The referees' comments are appended below, or you can access them online: please go to BenchPressand click on the 'Manuscripts with Decisions' queue in the Author Area.

As you will see, the referees express considerable interest in your work, but have some significant criticisms and recommend a revision of your manuscript before we can consider publication. If you are able to revise the manuscript along the lines suggested, which may involve further experiments, I will be happy receive a revised version of the manuscript. Your revised paper will be re-reviewed by one or more of the original referees, and acceptance of your manuscript will depend on your addressing satisfactorily the reviewers' major concerns. Please also note that Development will normally permit only one round of major revision. If it would be helpful, you are welcome to contact us to discuss your revision in greater detail. Please send us a point-by-point response indicating your plans for addressing the referee’s comments, and we will look over this and provide further guidance.

Please attend to all of the reviewers' comments and ensure that you clearly highlight all changes made in the revised manuscript. Please avoid using 'Tracked changes' in Word files as these are lost in PDF conversion. I should be grateful if you would also provide a point-by-point response detailing how you have dealt with the points raised by the reviewers in the 'Response to Reviewers' box. If you do not agree with any of their criticisms or suggestions please explain clearly why this is so.

© 2023. Published by The Company of Biologists under the terms of the Creative Commons Attribution License (https://creativecommons.org/licenses/by/4.0/).

Reviewer 1

Advance summary and potential significance to field

This manuscript provides the most extensive investigation of mouse placental polyploidy to date. A major finding of this manuscript is the much greater extent of placental polyploidy than previously anticipated. Further, the authors show a conserved role for myc in endocycles/polyploidy. Overall, these findings are of high impact as they highlight a much greater extent of mammalian polyploidy than previously anticipated, a finding with clear implications for fertility and reproduction.

Comments for the author

1. Results: “All together these data are the first to demonstrate widespread polyploidy in murine placental cell types via endocycles”

While your data support widespread polyploidy, endocycles vs. endomitosis vs. cell fusion are not carefully examined in this study. There is no examination of the transition from diploid to polyploid, during which one could study the precise mechanism. Please revise or provide additional supporting evidence. I agree that endocycles are the most likely if the cells have mononucleate polyploidy, but even there are also cases of multiple nuclei forming one larger nuclei during a successive cell cycle.

2. Figure 1: the DNA FISH pattern is interesting. To support the conclusions the authors should provide data showing a second DNA FISH probe, to assess the generality of their findings.

3. Figure 1: “This loose association and heterogeneity is distinct from Drosophila polytene chromosomes in the salivary gland which form more homogeneous tight associations (Stormo and Fox, 2017).”

This is correct, but there are also other examples in the literature that are consistent with FISH signals shown here, such as in non-polytene polyploid cells such as Drosophila nurse cells (Dej and Spradling 199) and rectal papillar cells (Stormo and Fox 2016). Further the satellite DNA signals in TGCs in Figure 1 of Varmuza et al (1988) also show a similar pattern. Please acknowledge these findings.

4. Figure 2: Is cell number different in c-myc embryos? Please provide some quantitation of this. If cell number is impacted, this would argue that myc is a general regulator of both mitotic and endocycles in the placenta.

5. Fig2F- the quality of the FISH images is not as good as in Fig1- how did you count distinct spots? This raises concerns about the FISH data in this figure though the integrated density data seem to support the overall conclusion about myc/ploidy. Please address the quality of the FISH data.

6. Typo, page 7- “Figure 3E” should be “Figure 2E”

7. Figure 5- the conclusion that c-MYC is required for rRNA expression in TGCs seems well supported. But whether rRNA expression is preferentially altered relative to many other mRNAs is unclear, as is the direct nature of c-MYC’s impact on rRNA expression. The authors should discuss this and also cite the Drosophila study by Grewal et al (2005) “Myc-dependent regulation of ribosomal RNA synthesis during Drosophila development.”

8. General comment- the claims about your finding of the most polyploid organ in the pregnant female mouse- the problem is your study shows that ploidy has not been exhaustively studied in the mouse. Rather, your study highlights the need to look for additional polyploid cell types. Along those lines, a relevant paper to cite would be the new polyploid reporter mouse generated in Matsumoto et al 2020.

9. Several citations should be added to the manuscript regarding prior work on polyploidy in the placenta. These include:

© 2023. Published by The Company of Biologists under the terms of the Creative Commons Attribution License (https://creativecommons.org/licenses/by/4.0/).

Gerbaud and Pidoux 2015
Soygur and Sati 2016
Simpson et al 1992
Ullah et al 2008
Ullah et al 2009
Ullah et al 2018
and there is decades of work on this topic by TG and EV Zybina that should be acknowledged

10. Several citations should be added to the manuscript regarding prior work on myc/polyploidy. These include:
Zhou F et al 2020
Grendler et al 2019
Scherbata et al 2004
Sargent et al 1996

11. Discussion- please address possible mechanisms of DNA breaks in c-MYC-/-placental cells. Also, in Drosophila, under-replication of DNA causes increased breaks in polyploid cells, and under-replication is also reported in the placenta so one might have expected the opposite result.

12. Figure legends- I could not find any information on the meaning of the number of * in each graph (what is the p value?) and also could not find information on the statistical test used in each case. Also, in addition to providing the number of cells/placentas examined, it would be valuable to provide information on the number of biological replicates.

Reviewer 2

Advance summary and potential significance to field

Overall this is an interesting and overdue study examining the basis for the c-Myc mutant placental insufficiency phenotype identified by Dubois et al. (2008). The present manuscript significantly extends the findings of Dubois et al., and its novelty is in no way diminished by this prior work. While Dubois et al. is cited, clearer acknowledgement of the placental phenotypes reported by Dubois et al. would have been welcome and appropriate in this manuscript.

The authors analyse ploidy across a range of placental cell types using 5S rDNA FISH, and present some interesting findings that cell types that may have previously been assumed to be diploid are in fact polyploid.

scRNA-seq comparisons of diploid vs polyploid cells suggest a role for polyploid cells in inflammation. The authors show that a group of polyploid cells activate c-Myc, and that c-Myc is a regulator of placental ploidy, and inflammation. They further show that loss of c-Myc results in genomic instability and senescence.

Overall they provide important new knowledge concerning placental development, and highlight a likely conserved role for c-Myc in the regulation of polyploidy. The experiments appear to be well-controlled and the data are largely convincing. However, clarifications are needed for some experiments. There also appear to have been some missed opportunities. These are detailed below. However, overall this is an interesting study with clear relevance to a fundamental developmental biology question. It is also potentially relevant to the evolutionary and pathological control of endoreduplication, as the authors point out.

Comments for the author

1. Figure 1B shows multiple different cell types 5S rDNA FISH for multiple cell types. How were these cell types identified in the histological sections. For example, how can you be confident that the cells stated to by GlyTs are GlyTs without reference to specific markers? Why were the cell types shown in Figure 1B,C chosen and others omitted e.g. canal TGCs, SpA-TGCs and other trophoblast subtypes. At 14.5 dpc the identity of these cells is likely to be fairly clear in histological sections so it seems a missed opportunity not to have analysed these also. Similarly, would it not

© 2023. Published by The Company of Biologists under the terms of the Creative Commons Attribution License (https://creativecommons.org/licenses/by/4.0/).

have been interesting to see whether syncytiotrophoblast nuclei are diploid? Please clarify how cells were identified, provide additional analyses if possible, and otherwise acknowledge the range of cell types not analysed.

2. Related to this – page 4, para 2 “Surprisingly, the majority of placental cell types, including sinusoidal trophoblast giant cells (S-TGCs), spongiotrophoblasts (SpTs), and glycogen cells (GlyTs) had ploidy >8C based on multiple 5S foci (Figure 1B, C).” This is an overinterpretation since the majority of placental cell types are arguably not represented in Figure 1B,C (see above comment).

3. Identification of polyploid regulators mentioned on page 5/6 appears to be based on comparison of a single presumably diploid cell type (hematopoietic cells) to multiple polyploid cell types. It is not clear to me that this is a wholly appropriate comparison. How do we know that the differences are not driven by the choice of diploid cell type rather than the fact that they are diploid. Were there not other diploid cell types within the dataset (e.g. endothelial cells), or cells that are polyploid due to cell fusion rather than endoreduplication (i.e. SynTs) that would have allowed a more complete and appropriate comparison here?

Please justify your approach, and perform an enhanced analysis if possible.

4. How many independent scRNA-seq experiments were performed? I am not suggested further experiments be performed, but I do think the number should be reported.

5. Page 7, para 2 – Figure 3E is cited when Figure 2E is meant. Also, the 5S rDNA FISH signal is hard to see in this panel compared to Figure 1B. Can the signal be made clearer?

6. Page 7, para 3 – More detail on how the TGC layer was dissected would be appreciated. Which TGCs and from where? I can see that a reference is provided in the Methods, but given the importance of the approach it should ideally be clearer in the manuscript.

7. Figure 3 – the key referring to “UP” and “DOWN” could be made clearer by increasing the font size and stating if which samples gene expression is up or down. Also, it would be clearer if the colour coding of panel C matched panel B.

8. Page 8, para 1 – “c-Myc mRNA levels were reduced 5- fold in the null compared to wild type, confirming the genotype.” – this should be shown.

9. Page 9 – please consider whether Figure S4 should be a main figure.

10. Page 10, para 3 – there are fewer nascent rRNA transcripts in c-Myc null TGCs, and shrunken nucleoli. However, ploidy is also reduced. Would we not therefore expect fewer nascent rRNA transcripts, and shrunken nucleoli? Some further discussion of these results and cause and effect would be appreciated in the Discussion.

11. Figure S6A,B – it appears to be a strange omission that there is no TGC image in the figure but TGCs are in the boxplot.

12. Page 11, para 2 – “Treatment with BMH21 causes the expected nucleolar stress phenotype (Figure S6C).” It is not clear to me what this expected phenotype is, or what I am meant to have seen in the figure. Please clarify.

13. Page 11, para 3 – “We provide evidence that many mature mouse placental cell types are polyploid via an endocycling mechanism, making the murine placenta the organ with the most polyploid cell types in a pregnant female.” I feel that this overstates the results of the work. No comparison is drawn with other organs, but I suspect that most researchers in this field would have already understood that there are more polyploid cells in the mouse placenta than other tissues and organs. Furthermore, the ploidy of many of the trophoblast (and other) cell types in the placenta were not analysed in this study, as stated above. Toning down the language a little and acknowledging the deficiencies of the study would therefore be appropriate.

© 2023. Published by The Company of Biologists under the terms of the Creative Commons Attribution License (https://creativecommons.org/licenses/by/4.0/).

14. A further analysis of the cell types affected in c-Myc mutants would have been interesting. For example, are syncytiotrophoblasts affected/lost? They appear to be in Dubois et al. (2008), but this has not been clearly established. Nor has the point at which defects in c-Myc mutant extraembryonic tissues occur. As such, a comprehensive analysis of the c-Myc mutant placental phenotype is still lacking. The authors do not claim that they have performed this, and the present study still represents a clear advance. A few statements in the discussion detailing the outstanding questions would be welcome.

Minor points:

1. Page 3, para 1 – "It performs several vital functions such as nutrient transport, hormone production, hematopoiesis, and most importantly it protects the fetus from the immunological response of the mother." I would not state "most importantly". Protecting the fetus from the maternal immune system is irrelevant if the placenta cannot perform its other functions. Also, it would have been easier for the reviewer to refer to specific pages and sentences if page and line numbers had been included in the manuscript.

2. Page 4, para 2 – "The polyploidy of primary trophoblast giant cells (P-TGCs) and syncytiotrophoblasts has been established" – I am concerned that parietal TGCs (P-TGCs) and primary TGCs are conflated in this manuscript. Some clarification would be appreciated.

3. Page 5, para 2 – "We were able to identify 31 clusters (Figure S1A)." Figure S1A appears to show 34 clusters.

4. Figure S3A – the key could be larger.

5. Thickness of histological sections are referred to in millimetres throughout when I think micrometres is meant. Please correct in all locations.

6. Page 10, line 1 – I am not familiar with ONPG. This seems like technical detail that belongs in the Methods. Otherwise, please explain in context.

7. Page 11, para 1 – "gH2A.X" – did you intend to use the gamma symbol instead of g?

First revision

Author response to reviewers' comments

We sincerely thank the editor and reviewers for suggestions to improve our manuscript. We have revised the manuscript and below is a brief summary of changes, followed by a point by point response. We believe the manuscript is now ready for publication.

1. We added the references requested and modified the discussion accordingly.
2. We amended claims regarding polyploidy to reflect that it is an understudied state and more examples are likely to be discovered, rather than claiming the placenta is the organ with the highest number of polyploid cells.
3. We used TGC nomenclature more clearly and consistently.
4. We added Figure S1, which includes a FISH probe to single copy gene Prl8a8, to further demonstrate the polyploidy of TGCs.
5. We added one more diploid cell type, monocytes, to the IPA analysis (Figure 1E) to identify regulators of polyploidy.
6. In Figure 2F we added a quantification of the number of TGCs in the c-Myc mutant compared to WT.
7. We have added new images to Figure 2 G,H to show ploidy of c-Myc null TGCs.
8. Figure 3A was amended to display the method of TGC dissection and to color code the up and down regulated genes (Figure 3 B,C).

© 2023. Published by The Company of Biologists under the terms of the Creative Commons Attribution License (https://creativecommons.org/licenses/by/4.0/).

9. We moved Figure S5 into the main manuscript as Figure 5.
10. In Figure 6 we added quantification of the mean nuclear intensity of γH2AX.
11. We added representative images of TGCs to Figure S6A.

Point wise response to reviewer's comments:

Reviewer 1
Advance summary and potential significance to field
This manuscript provides the most extensive investigation of mouse placental polyploidy to date. A major finding of this manuscript is the much greater extent of placental polyploidy than previously anticipated. Further, the authors show a conserved role for myc in endocycles/polyploidy. Overall, these findings are of high impact as they highlight a much greater extent of mammalian polyploidy than previously anticipated, a finding with clear implications for fertility and reproduction.

Our reply: We thank reviewer for appreciating our work and providing important feedback to improve our manuscript.

Reviewer 1 Comments for the author

1.Results: "All together these data are the first to demonstrate widespread polyploidy in murine placental cell types via endocycles". While your data support widespread polyploidy, endocycles vs. endomitosis vs. cell fusion are not carefully examined in this study. There is no examination of the transition from diploid to polyploid, during which one could study the precise mechanism. Please revise or provide additional supporting evidence. I agree that endocycles are the most likely if the cells have mononucleate polyploidy, but even there are also cases of multiple nuclei forming one larger nuclei during a successive cell cycle.

Our reply: We agree with the reviewer. We have edited the manuscript to suggest that endocyling could be the mechanism but acknowledging further research is warranted.

2.Figure 1: the DNA FISH pattern is interesting. To support the conclusions, the authors should provide data showing a second DNA FISH probe, to assess the generality of their findings.

Our reply: We have added two additional DNA FISH probes; one single copy gene probe to Prl8a8 and a second 45S rRNA gene which is on 5 different chromosomes in multicopy arrays (Figure S1). In tissue sections we did not obtain good signal from the single copy gene probe in diploid cells but P-TGCs showed the same pattern as we observe with the 5S DNA FISH probe, validating the finding in P-TGCs. Because 45S rDNA is on 5 chromosomes in the mouse genome, we could not observe separate foci and this probe was not useful to quantify ploidy. In P-TGCs, 5S and Prl8a8 DNA-FISH results are similar to Varmuza et al (1988), which further strengthens our results. We acknowledge that more work will be required to understand polyploidy in various placental cell types.

3.Figure 1: "This loose association and heterogeneity is distinct from Drosophila polytene chromosomes in the salivary gland which form more homogeneous tight associations (Stormo and Fox, 2017)." This is correct, but there are also other examples in the literature that are consistent with FISH signals shown here, such as in non-polytene polyploid cells such as Drosophila nurse cells (Dej and Spradling 1999) and rectal papillar cells (Stormo and Fox 2016). Further the satellite DNA signals in TGCs in Figure 1 of Varmuza et al (1988) also show a similar pattern. Please acknowledge these findings.

Our reply: We have added these references and highlighted these points in the results.

4.Figure 2: Is cell number different in c-myc embryos? Please provide some quantitation of this. If cell number is impacted, this would argue that myc is a general regulator of both mitotic and endocycles in the placenta.

Our reply: We were not sure if the reviewer meant to ask about embryos or P-TGCs. We now report that the number of P-TGCs in the null is significantly lower than WT(Figure 2F). We did not attempt to quantify the number of cells in the embryo although embryos from the cross were small in size, suggesting that myc is needed for proliferation, now noted in the manuscript. However, we believe

© 2023. Published by The Company of Biologists under the terms of the Creative Commons Attribution License (https://creativecommons.org/licenses/by/4.0/).

this is due to the underdeveloped placenta. In the previous report using Sox2-cre to knockout Myc in embryos (Dubois et al., 2008), embryos were similar in size to WT up to e10.5. Close to their time of death at e11.5 mutant embryos are slightly smaller and pale due to hematopoetic deficiency. This is now better explained in the manuscript.

5.Fig2F- the quality of the FISH images is not as good as in Fig1- how did you count distinct spots? This raises concerns about the FISH data in this figure, though the integrated density data seem to support the overall conclusion about myc/ploidy. Please address the quality of the FISH data.

Our reply: These (Old Figure 2F, now Figure 2G) DNA-FISH images are on Cryo sections to stain TGCs with PLF antibody whereas Figure 1B images are on Paraffin sections. We agree that Cryo sections have more background as compared to Paraffin sections-its simply a reflection of the method. We have improved the quality of images for Figure 2G but due to the method the images are not as good.

6.Typo, page 7- “Figure 3E” should be “Figure 2E”

Our reply: We have amended this error.

7.Figure 5- the conclusion that c-MYC is required for rRNA expression in TGCs seems well supported. But whether rRNA expression is preferentially altered relative to many other mRNAs is unclear, as is the direct nature of c-MYC’s impact on rRNA expression. The authors should discuss this and also cite the Drosophila study by Grewal et al (2005) “Myc-dependent regulation of ribosomal RNA synthesis during Drosophila development.”

Our reply: We agree that it is difficult to differentiate between direct and indirect effects. There are published papers, including the one pointed out by the reviewer, arguing that Myc is a transcription factor for RNAP1. We acknowledge in the manuscript that we have not performed experiments to differentiate between direct and indirect effects of Myc removal, but note that our results are consistent with other reports that c-Myc can regulate RNA Pol I. We have added a citation to the Grewal et al (2005) study.

8.General comment- the claims about your finding of the most polyploid organ in the pregnant female mouse- the problem is your study shows that ploidy has not been exhaustively studied in the mouse. Rather, your study highlights the need to look for additional polyploid cell types. Along those lines, a relevant paper to cite would be the new polyploid reporter mouse generated in Matsumoto et al 2020.

Our reply: We have amended the discussion to highlight that more exploration of polyploidy and its mechanisms are warranted, and added the reference suggested.

9.Several citations should be added to the manuscript regarding prior work on polyploidy in the placenta. These include:
Gerbaud and Pidoux 2015
Soygur and Sati 2016
Simpson et al 1992
Ullah et al 2008
Ullah et al 2009
Ullah et al 2018
and there is decades of work on this topic by TG and EV Zybina that should be acknowledged

Our reply: We have added these references.

10.Several citations should be added to the manuscript regarding prior work on myc/polyploidy. These include:
Zhou F et al 2020
Grendler et al 2019
Scherbata et al 2004
Sargent et al 1996

© 2023. Published by The Company of Biologists under the terms of the Creative Commons Attribution License (https://creativecommons.org/licenses/by/4.0/).

Our reply: We have added these references except for Sargent et al which is primarily focused on aneuploidy (not polyploidy) in murine Myc-transgenic livers.

11.Discussion- please address possible mechanisms of DNA breaks in c-MYC-/- placental cells. Also, in Drosophila, under-replication of DNA causes increased breaks in polyploid cells, and under-replication is also reported in the placenta, so one might have expected the opposite result.

Our reply: We agree this could have gone either way, that was why we decided to check for DNA damage! The increased DNA damage in the absence of Myc is most consistent with replication stress. We posit that c-MYC normally promotes expression of genes for nucleotide biosynthesis and DNA replication, and their reduced expression leads to replication stress, and increased DNA damage. Inhibiting rRNA synthesis with BMH21 does NOT have this affect, consistent with our suggestion that the defect arises from gene expression based insufficiency of replication and nucleotide biosynthesis factors. We have made these points more explicit.

12.Figure legends- I could not find any information on the meaning of the number of * in each graph (what is the p value?) and also could not find information on the statistical test used in each case. Also, in addition to providing the number of cells/placentas examined, it would be valuable to provide information on the number of biological replicates.

Our reply: We have added this information to the figure legends. * $p<0.05$, **$p<0.01$, ***$p<0.001$, and ****$p<0.0001$.

Reviewer 2
Advance summary and potential significance to field
Overall this is an interesting and overdue study examining the basis for the c-Myc mutant placental insufficiency phenotype identified by Dubois et al. (2008). The present manuscript significantly extends the findings of Dubois et al., and its novelty is in no way diminished by this prior work. While Dubois et al. is cited, clearer acknowledgement of the placental phenotypes reported by Dubois et al. would have been welcome and appropriate in this manuscript.

The authors analyse ploidy across a range of placental cell types using 5S rDNA FISH, and present some interesting findings that cell types that may have previously been assumed to be diploid are in fact polyploid. scRNA-seq comparisons of diploid vs polyploid cells suggest a role for polyploid cells in inflammation. The authors show that a group of polyploid cells activate c-Myc, and that c-Myc is a regulator of placental ploidy, and inflammation. They further show that loss of c-Myc results in genomic instability and senescence. Overall they provide important new knowledge concerning placental development, and highlight a likely conserved role for c-Myc in the regulation of polyploidy. The experiments appear to be well-controlled and the data are largely convincing. However, clarifications are needed for some experiments. There also appear to have been some missed opportunities. These are detailed below. However, overall this is an interesting study with clear relevance to a fundamental developmental biology question. It is also potentially relevant to the evolutionary and pathological control of endoreduplication, as the authors point out.

Our reply: We thank the reviewer for providing important input to improve our manuscript. We agree that more description of the findings in Dubois et al. help with the interpretation and contextualization of the current findings and we have expanded our description of Dubois et al..

Reviewer 2
Comments for the author
1.Figure 1B shows multiple different cell types 5S rDNA FISH for multiple cell types. How were these cell types identified in the histological sections. For example, how can you be confident that the cells stated to by GlyTs are GlyTs without reference to specific markers? Why were the cell types shown in Figure 1B,C chosen and others omitted e.g. canal TGCs, SpA-TGCs and other trophoblast subtypes. At 14.5 dpc the identity of these cells is likely to be fairly clear in histological sections so it seems a missed opportunity not to have analysed these also. Similarly, would it not have been interesting to see whether syncytiotrophoblast nuclei are diploid? Please clarify how cells were identified, provide additional analyses if possible, and otherwise acknowledge the range of cell types not analysed.

© 2023. Published by The Company of Biologists under the terms of the Creative Commons Attribution License (https://creativecommons.org/licenses/by/4.0/).

Our reply: We identify cells based on morphology and spatial positions, therefore only certain cell types can be analyzed. It was difficult to unequivocally identify canal-TGCs, SpA-TGCs and syncytiotrophoblast in DAPI staining without using specific markers. Most antibodies are not compatible with the DNA FISH protocol, so adding markers was not possible. We edited the manuscript to explain the rationale in more detail and acknowledge limitations.

2.Related to this – page 4, para 2 “Surprisingly, the majority of placental cell types, including sinusoidal trophoblast giant cells (S-TGCs), spongiotrophoblasts (SpTs), and glycogen cells (GlyTs) had ploidy >8C based on multiple 5S foci (Figure 1B, C).” This is an overinterpretation since the majority of placental cell types are arguably not represented in Figure 1B,C (see above comment).

Our reply: Thank you for this point. We have modified this statement accordingly.

3.Identification of polyploid regulators mentioned on page 5/6 appears to be based on comparison of a single presumably diploid cell type (hematopoietic cells) to multiple polyploid cell types. It is not clear to me that this is a wholly appropriate comparison. How do we know that the differences are not driven by the choice of diploid cell type rather than the fact that they are diploid. Were there not other diploid cell types within the dataset (e.g. endothelial cells), or cells that are polyploid due to cell fusion rather than endoreduplication (i.e. SynTs) that would have allowed a more complete and appropriate comparison here? Please justify your approach, and perform an enhanced analysis if possible.

Our reply: We have enhanced our analysis to include one more diploid cell type (Monocytes) along with hematopoietic stem cells and the results are similar. We included endothelial cells and SynTs cells in our previous analysis, but we were unsure about their ploidy. For endothelial cells it has been suggested that with age tetraploidy is common in these cells and placenta at 14.5 dpc is fully developed so there is high chance of polyploid endothelial cells (W. W. Nichols et. al. 1987, Cytogenetic evaluation of human endothelial cell cultures).

4.How many independent scRNA-seq experiments were performed? I am not suggested further experiments be performed, but I do think the number should be reported.

Our reply: Two independent scRNA-seq experiment was performed and we have added this information clearly in methods and figure legends.

5.Page 7, para 2 – Figure 3E is cited when Figure 2E is meant. Also, the 5S rDNA FISH signal is hard to see in this panel compared to Figure 1B. Can the signal be made clearer?

Our reply: These (Old Figure 2F, now Figure 2G) DNA-FISH images were performed on Cryo sections to stain TGCs with the anti-PLF antibody whereas Figure 1B images are derived from Paraffin sections. Cryo sections have more background as compared to Paraffin section. We have improved the quality of images for Figure 2G but they are still less clear than 1B due to the difference in methodology.

6.Page 7, para 3 – More detail on how the TGC layer was dissected would be appreciated. Which TGCs and from where? I can see that a reference is provided in the Methods, but given the importance of the approach it should ideally be clearer in the manuscript.

Our reply: We have added this information in Figure 3A.

7.Figure 3 – the key referring to “UP” and “DOWN” could be made clearer by increasing the font size and stating if which samples gene expression is up or down. Also, it would be clearer if the colour coding of panel C matched panel B.
Our reply: We have updated this image and added matched color coding to Figure 3B,C.

8.Page 8, para 1 – “c-Myc mRNA levels were reduced 5- fold in the null compared to wild type, confirming the genotype.” – this should be shown.

Our reply: We have highlighted c-Myc in the volcano plot (Figure 3B) as requested.

© 2023. Published by The Company of Biologists under the terms of the Creative Commons Attribution License (https://creativecommons.org/licenses/by/4.0/).

9.Page 9 – please consider whether Figure S4 should be a main figure.

Our reply: Figure S4 is now Figure 5 in the revised manuscript, as suggested.

10.Page 10, para 3 – there are fewer nascent rRNA transcripts in c-Myc null TGCs, and shrunken nucleoli. However, ploidy is also reduced. Would we not therefore expect fewer nascent rRNA transcripts, and shrunken nucleoli? Some further discussion of these results and cause and effect would be appreciated in the Discussion.

Our reply: We have quantified nascent rRNA transcription as mean intensity, which is total integrated density divided by area. This should normalize for the reduced ploidy in c-Myc null P-TGCs. To support the claim that nucleolar morphology can reflect nucleolar stress, we have added a reference and we explain the similarity to the nucleolar phenotype with BMH21 treatment (RNAP1 inhibition).

11.Figure S6A,B – it appears to be a strange omission that there is no TGC image in the figure but TGCs are in the boxplot.

Our reply: We have added an image of P-TGCs in the revised Figure S6A.

12.Page 11, para 2 – “Treatment with BMH21 causes the expected nucleolar stress phenotype (Figure S6C).” It is not clear to me what this expected phenotype is, or what I am meant to have seen in the figure. Please clarify.

Our reply: When RNAP1 transcription is inhibited, nucleoli shrink and round up. This is now explained and we have cited a reference that details how different nucleolar morphologies are characteristic of different types of stress.

13.Page 11, para 3 – “We provide evidence that many mature mouse placental cell types are polyploid via an endocycling mechanism, making the murine placenta the organ with the most polyploid cell types in a pregnant female.” I feel that this overstates the results of the work. No comparison is drawn with other organs, but I suspect that most researchers in this field would have already understood that there are more polyploid cells in the mouse placenta than other tissues and organs. Furthermore, the ploidy of many of the trophoblast (and other) cell types in the placenta were not analysed in this study, as stated above. Toning down the language a little and acknowledging the deficiencies of the study would therefore be appropriate.

Our reply: We agree with the reviewer’s point and we have modified these statements.

14.A further analysis of the cell types affected in c-Myc mutants would have been interesting. For example, are syncytiotrophoblasts affected/lost? They appear to be in Dubois et al. (2008), but this has not been clearly established. Nor has the point at which defects in c-Myc mutant extraembryonic tissues occur. As such, a comprehensive analysis of the c-Myc mutant placental phenotype is still lacking. The authors do not claim that they have performed this, and the present study still represents a clear advance. A few statements in the discussion detailing the outstanding questions would be welcome.

Our reply: We agree with reviewer’s points. We have added a statement to the discussion that much remains to be learned regarding additional cell types and polyploidy in the placenta and beyond.

Minor points:
1.Page 3, para 1 – “It performs several vital functions such as nutrient transport, hormone production, hematopoiesis, and most importantly it protects the fetus from the immunological response of the mother.” I would not state “most importantly”. Protecting the fetus from the maternal immune system is irrelevant if the placenta cannot perform its other functions. Also, it would have been easier for the reviewer to refer to specific pages and sentences if page and line numbers had been included in the manuscript.

Our reply: We have modified this sentence. We added page numbers.

© 2023. Published by The Company of Biologists under the terms of the Creative Commons Attribution License (https://creativecommons.org/licenses/by/4.0/).

2.Page 4, para 2 – "The polyploidy of primary trophoblast giant cells (P-TGCs) and syncytiotrophoblasts has been established" – I am concerned that parietal TGCs (P-TGCs) and primary TGCs are conflated in this manuscript. Some clarification would be appreciated.

Our reply: We focused on parietal TGCs (P-TGCs) which includes both primary and secondary TGCs. We now use consistent nomenclature throughout the manuscript.

3.Page 5, para 2 – "We were able to identify 31 clusters (Figure S1A)." Figure S1A appears to show 34 clusters.

Our reply: Yes, there should be 34 clusters in Figure S2A (new). We have updated this information.

4.Figure S3A – the key could be larger.

Our reply: We have modified Figure S4B to include a larger key.

5.Thickness of histological sections are referred to in millimetres throughout when I think micrometres is meant. Please correct in all locations.

Our reply: Yes, this is true and now we have carefully corrected it.

6.Page 10, line 1 – I am not familiar with ONPG. This seems like technical detail that belongs in the Methods. Otherwise, please explain in context.

Our reply: We have added about the full chemical name of ONPG and that it is a chromogenic substrate for b-gal.

7.Page 11, para 1 – "gH2A.X" – did you intend to use the gamma symbol instead of g?

Our reply: It should be gamma and we have changed it.

Second decision letter

MS ID#: DEVELOP/2023/201581

MS TITLE: c-Myc promotes polyploidy in murine trophoblast cells and suppresses senescence

AUTHORS: Vijay Pratap Singh, Huzaifa Hassan, Fengyan Deng, Dai Tsuchiya, Sean McKinney, Kevin Ferro, and Jennifer Gerton
ARTICLE TYPE: Research Article

I am happy to tell you that your manuscript has been accepted for publication in Development, pending our standard ethics checks.

Reviewer 1

Advance summary and potential significance to field

I echo my previous comments- this is an important paper for the field as it reveals new roles and regulation for polyploidy in development.

Comments for the author

The revised manuscript addresses all of my previous comments. Well done!

© 2023. Published by The Company of Biologists under the terms of the Creative Commons Attribution License (https://creativecommons.org/licenses/by/4.0/).

Reviewer 2

Advance summary and potential significance to field

I am satisfied with the revisions and am happy to endorse this article for publication.

Comments for the author

I have no further suggestions.

© 2023. Published by The Company of Biologists under the terms of the Creative Commons Attribution License (https://creativecommons.org/licenses/by/4.0/).
